# A systematic review of the profile and density of the maternal and child health workforce in China

**DOI:** 10.1186/s12960-021-00662-4

**Published:** 2021-10-09

**Authors:** Huan Zhang, Xiaoyun Liu, Loveday Penn-Kekana, Carine Ronsmans

**Affiliations:** 1 Department of Infectious Disease Epidemiology, London School of Hygiene and Tropical Medicine, WC1E 7HT London, United Kingdom; 2 Peking University China Centre for Health Development Studies, Beijing, China

**Keywords:** Human resources for health, Maternal and child health, China, Systematic review

## Abstract

**Background:**

To track progress in maternal and child health (MCH), understanding the health workforce is important. This study seeks to systematically review evidence on the profile and density of MCH workers in China.

**Methods:**

We searched 6 English and 2 Chinese databases for studies published between 1 October 1949 and 20 July 2020. We included studies that reported on the level of education or the certification status of all the MCH workers in one or more health facilities and studies reporting the density of MCH workers per 100 000 population or per 1000 births. MCH workers were defined as those who provided MCH services in mainland China and had been trained formally or informally.

**Results:**

Meta-analysis of 35 studies found that only two-thirds of obstetricians and paediatricians (67%, 95% CI: 59.6–74.3%) had a bachelor or higher degree. This proportion was lower in primary-level facilities (28% (1.5–53.9%)). For nurses involved in MCH care the proportions with a bachelor or higher degree were lower (20.0% (12.0–30.0%) in any health facility and 1% (0.0–5.0%) in primary care facilities). Based on 18 studies, the average density of MCH doctors and nurses was 11.8 (95% CI: 7.5–16.2) and 11.4 (7.6–15.2) per 100 000 population, respectively. The average density of obstetricians was 9.0 (7.9–10.2) per 1000 births and that of obstetric nurses 16.0 (14.8–17.2) per 1000 births. The density of MCH workers is much higher than what has been recommended internationally (three doctors and 20 midwives per 3600 births).

**Conclusions:**

Our review suggests that the high density of MCH workers in China is achieved through a mix of workers with high and low educational profiles. Many workers labelled as “obstetricians” or “paediatrician” have lower qualifications than expected. China compensates for these low educational levels through task-shifting, in-service training and supervision.

**Supplementary Information:**

The online version contains supplementary material available at 10.1186/s12960-021-00662-4.

## Background

Maternal and child mortality levels have fallen substantially in China since the Liberation in 1949. China’s maternal mortality ratio was estimated to be around 1500 deaths per 100 000 live births in 1950 [1], dropping to 17.8 in 2019[2]. The under-five mortality rate dropped from 210.7 to 7.8 deaths per 1000 live births over the same period [1, 2]. Investment in health systems has no doubt accelerated the progress in reducing maternal and child mortality [3, 4]. Key to this success has been the training and deployment of maternal and child health (MCH) workers, which are seen as the cornerstone of successful MCH programmes [5]. Understanding who they are, and how many (per unit of population), is critical to the planning of such services.

Defining what constitutes an “MCH worker”, and what their qualifications should be, remains a challenge internationally as well as in China. A joint report issued by the World Health Organization (WHO) and the United Nations Population Fund, defined sexual, reproductive, maternal, newborn and adolescent health workers as those including but not limited to “midwives, nurses, nurse-midwives, general practitioners, specialist doctors (such as obstetrician/gynaecologist, neonatologists, paediatricians), auxiliary staff, community health workers, and support workers (including traditional birth attendants)” [6]. The joint report did not define the health workers by cadre, and advised national workforce assessments to categorize health workers according to the cadre titles used in their own country. However, the full complexity and dynamics of the MCH workforce within each country is difficult to capture. So far, most of the research in this area has focused on the definition and measurement of particular cadres of MCH workers, e.g. midwives or paediatricians, not the entire workforce [7–12]. The WHO, for example, has recently updated its definition of a skilled birth attendant [13], but that definition does not necessarily help with human resource planning, since the criteria used are not well aligned with in-country training or qualification systems.

In China, there is no consensus on what constitutes an MCH worker. The health system allows extensive variation in education, roles and responsibilities between health workers, and definitions vary over time and between geographical locations [14]. Terms such as “obstetrician” or “paediatrician” are used loosely, and there is no standard definition. To our knowledge, there has not been a comprehensive assessment of the profile of MCH workers in China.

Education level, health-related discipline and certification are most commonly used to understand the profile of health workers in China [14–16]. Education levels generally move from primary or middle school to high school or secondary technical school, junior college, Bachelor’s degree, Master’s degree, and Doctoral degree [14]. The length of medical training varies between different disciplines, ranging from three to eight years. The tracking of health-related disciplines is also important, because China’s educational reforms in 1998 redesigned medical training from the former Soviet model, which was based on empirical clinical training to a Western model, where categorizations are based on disciplines from the natural sciences [15]. Certification, and in particular the “MCH care certificate” (*Muying baojian jishu hegezheng,* thereafter referred to as certification), is critical in allowing MCH workers to perform certain duties. All MCH workers who are directly involved in prenatal diagnosis, delivery care and termination of pregnancy need to be certified by law to be allowed to perform these tasks [17]. To be eligible for a certificate, applicants need to have graduated from secondary technical schools or higher education and hold a valid medical doctor, nurse/midwife, or medical technician license. Candidates are certified by county-level or higher-level health authorities after passing a theoretical and skill examination, which is regulated by National Health and Family Planning Commission (previously the Ministry of Health).

The density of health workers is widely used as an indicator of health systems inputs [18, 19]. The Chinese government uses the “density of doctors per 1000 population” as a performance indicator, but there is very little information on the density of MCH workers. Work within the MCH field has suggested that the total population may not accurately reflect the obstetric or paediatric needs of a population. Maternal health worker density, for example, should be expressed over total number of births, while child health worker density should be counted per number of children [20–22]. This is particularly relevant for China since fertility rates are low [23]. Using births as the reference population, the World Health Report 2005 suggested a minimum requirement of 20 midwives or 3 doctors (at least part time) per 3600 births per year to ensure essential maternal and newborn care [24].

The lack of information on the profile and density of MCH workers in China greatly restricts evidence-informed policy making to address potential workforce issues. The aim of this study is to systematically review the literature reporting on the profile and density of MCH workers in mainland China.

## Methods

### Search strategy

We combined three search terms ‘human resources for health’, ‘MCH services’, and ‘China’ with both thesaurus and free-text words [see Additional file 1 for the full search strategy]. We searched six English databases (EMBASE, MEDLINE, The Cochrane Central Register of Controlled Trials, EconLit, Global Health and Web of Science) and two Chinese databases (China National Knowledge Infrastructure [CNKI] and Wanfang) with no limitation on language. We searched the literature between October 1, 1949 (founding of the People’s Republic of China), and July 20, 2020. We combined the search results and removed duplicate studies.

### Study selection

We screened studies based on information in their titles and abstracts. All potentially relevant studies were retrieved for the full texts and reviewed for inclusion. Studies were eligible for inclusion if they reported on MCH workers active in any aspect of MCH care from pregnancy to childhood, including antenatal care, childbirth care, postnatal care, care during infancy and care for children under five. MCH workers were defined as those who provided MCH services in mainland China and had been trained formally or informally. The services could be either preventive or curative: for example, vaccination or prescribing medication for diarrhoea. The studies could be peer-reviewed articles (English/Chinese) or Chinese Masters or Doctoral theses. We did not add reports published by the Chinese government such as the Health Statistics Yearbook, the National Health Survey Report, the National Survey of Health Resources and Medical Services, because these reports do not provide the profile, or the total count or the density of any cadre of MCH workers within a defined geographic area. Studies were excluded if published as conference abstract, poster or editorial.

For studies reporting on MCH workers’ profile, we included facility-based as well as population-based data, adding the following inclusion criteria: (1) studies needed to be based on a census or a sample of one or more cadres of MCH workers either from communities or from one or multiple units within one or multiple health facilities. Health facilities were defined as hospitals, health centres or clinics. (2) Reporting the numbers of at least one cadre of MCH workers, broken down by the level of education, health-related discipline or certification.

For studies reporting on the density of MCH workers, we only included population-based data, adding the following inclusion criteria: (1) the numerator was the whole count of any cadre of MCH workers in a population in a given geographical area, either from a population census with health occupation data, or from a health workforce survey with application of a sampling weight to calibrate for population representation. The denominator was either total population or specific subgroups such as total number of women, children or births for the same geographical area. (2) The studies either reported the density of any cadre of MCH workers directly or reported both the numerator and the denominator allowing us to calculate the density of MCH workers.

### Data extraction

We used a standard data extraction form in Excel to extract the following information: study source; study setting; data collection methods; MCH worker cadre and definition; MCH worker profile; MCH worker density. We compared author names, study setting, sampling methods, and time of study to detect duplicate studies.

### Risk of bias assessment

We used the component approach outlined in the Cochrane Handbook to assess the risk of bias of eligible studies [25]. We assessed the rigour of the study design (e.g. whether the sampling strategy for a survey was clearly described), the definition of the MCH workers and the completeness of data. We classified studies as having a low risk or high risk or unclear risk of bias. For example, a study was classified as having high risk of bias for study design if a cross-sectional survey in a single facility was claimed to be based on random sampling of MCH workers without information on the units from which the workers were sampled. The risk of bias was deemed to be unclear if the authors did not report the information for the above criteria.

### Data analysis

#### Profile of MCH workers

We grouped the education levels into three categories: bachelor or higher-level degree, junior college education, and secondary technical school or lower-level education. We categorized health facilities into three levels: tertiary (provincial- or municipal-level facilities), secondary (county-level facilities), and primary level (community health centres, township hospitals, village clinics).

We combined proportions of MCH workers by each education level using meta-analysis. To generate confidence intervals within admissible values, we used Clopper–Pearson exact method to compute the study specific confidence intervals. We performed the Freeman–Tukey double-arcsine transformation method to compute the weighted pooled estimates. In the subgroup meta-analyses, studies were stratified by level of health facility (tertiary/secondary/primary). Due to the variation between study characteristics, we used random-effects models in the meta-analysis to pool the proportions by each education level, presenting forest plots. We inspected I^2^ values and p values from the test of heterogeneity to assess evidence of between-study variation in the individual proportion estimates not due to random variation.

#### Density of MCH workers

For studies using total population as denominator, we converted the units of the density of MCH workers to per 100 000 population and presented the density by detailed cadre using forest plots. For studies using number of births as denominator, we summarized the studies in a forest plot. The method for the meta-analysis of density was exactly the same as what we did for the analysis on education level. Where data were available, we calculated the ratio of maternal health workers to child health workers or the ratio of doctors to nurses within individual studies.

All statistical analyses were performed using STATA (Version 14: Stata Corp).

## Results

### Description of the included studies

**Fig. 1:**
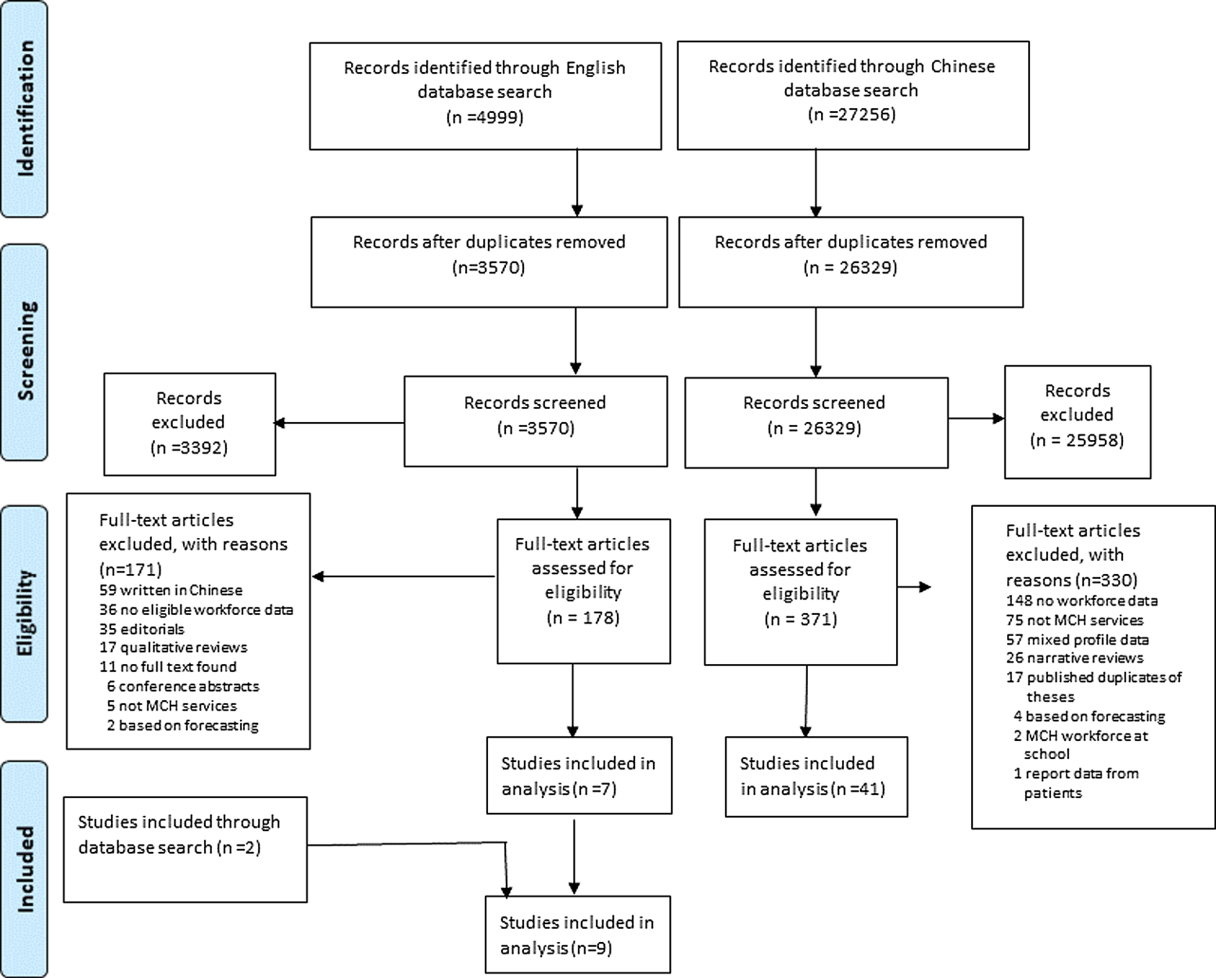
The flow diagram of study selection based on English and Chinese database searching (PRISMA 2009)

Figure 1 shows the results of the study search and selection. The database search identified 4999 English references and 27 256 Chinese references. After removing duplicates, 3392 English references and 25 958 Chinese references were excluded through title and abstract screening. We could not trace the full texts of 11 potentially relevant studies. Of the 178 English studies and 371 Chinese studies reviewed in full text, 48 studies were included. We reviewed the automatic e-mail updates of search results on a weekly basis until July 20, 2020, and added two English articles. We finally included a total of 50 studies: 35 reporting on MCH workforce profiles [16, 26–62], 18 reporting on the MCH workforce density [16, 53, 61, 63–77] and three covering both [16, 53, 61]. Most studies were peer-reviewed articles (*n* = 39, 78.0%) while the remaining were from Masters these (*n* = 11, 22.0%).

### Quality of included studies

For studies reporting on MCH workers’ profile, only eight were judged to be at low risk of bias across all the domains in the risk of bias assessment [33–35, 46, 48–50, 57]. For studies on MCH workforce’s density, all were judged to have unclear risk of bias [see Additional file 2 for quality assessment results].

### Studies reporting the profile of MCH workers

#### Study characteristics

Of the 35 studies, 33 (94.3%) were done after 1990, and 32 (91.4%) were done within a single province. Only one study was nationally representative, reporting on the education level of maternal and child health workers separately [16]. The MCH workforce in China covers an array of cadres, including obstetricians, gynaecologists, paediatricians, nurses, midwives, general practitioners, specialized public health workers, vaccinators, barefoot doctors, and traditional birth attendants [see Additional file 3]. Only two studies provided information on the certification held by MCH workers.

#### Education level

Education level was reported in nearly all the studies (n = 34, 97.1%). In the meta-analysis, the weighted average proportions of doctors having bachelor degree or above, junior college education, and secondary technical school or below were 67.0% (95% CI: 59.6–74.3%), 23.0% (10.0–40.0%), and 14.0% (5.0–28.0%), respectively (Table 1). The total was not exactly 100% because not all studies contributed data to each of the three categories. For example, a study may solely report the proportion of doctors holding bachelor degree or above and the aggregate proportion of junior college education or below, contributing only to the meta-analysis of the proportion of bachelor degree or above. For nurses, the estimated proportions were 20.0% (12.0–30.0%), 46.4% (41.2–51.5%) and 33.1% (24.2–41.9%), respectively. For other cadres, the estimated proportions were 18.0% (5.0–36.0%), 18.0% (6.0–34.0%) and 71.0% (46.0–91.0%), respectively.

**Table 1:** Meta-analyses of the proportion of education level, stratified by cadre and level of facility

	Bachelor or above		Junior college		Secondary technical school or below	
	Proportion, %	*I*^2^, %	Proportion, %	*I*^2^, %	Proportion, %	*I*^2^, %
Doctor						
Primary	27.7 (1.5–53.9)	97.2	47.0 (42.0–51.0)	89.8	41.0 (37.0–46.0)	98.7
Secondary	61.3 (31.7–90.9)	99.6	32.0 (10.0–59.0)	99.3	8.0 (1.0–20.0)	98.1
Tertiary	97.4 (94.9–100.0)	92.3	0.0 (0.0–2.0)	0.0	0.0 (0.0–1.0)	0.0
Mixed	65.1 (54.9–75.3)	99.7	29.0 (9.0–55.0)	99.1	34.0 (20.0–50.0)	96.1
Overall	67.0 (59.6–74.3)	99.8	23.0 (10.0–40.0)	99.6	14.0 (5.0–28.0)	99.1
Nurse						
Primary	1.0 (0.0–5.0)	–	40.0 (30.3–49.7)	–	59.0 (49.3–68.8)	–
Secondary	4.0 (2.7–5.3)	53.8	44.9 (35.4–54.4)	94.8	51.0 (42.9–59.2)	92.9
Tertiary	34.0 (20.9–50.0)	99.3	50.4 (38.5–62.2)	98.	23.7 (7.8–39.6)	99.2
Mixed	20.0 (9.0–33.0)	99.9	44.6 (38.0–51.1)	96.4	31.0 (20.1–42.0)	99.2
Overall	20.0 (12.0–30.0)	99.8	46.4 (41.2–51.5)	97.6	33.1 (24.2–41.9)	99.4
Other cadres						
Primary	3.0 (0.0–12.0)	96.4	11.1 (1.0–29.0)	98.1	83.0 (51.0–100.0)	99.7
Secondary	10.0 (3.0–19.0)	44.5	44.0 (37.0–52.0)	43.7	47.0 (32.0–62.0)	75.4
Tertiary	77.0 (58.0–91.0)	69.9	50.0 (18.7–81.3)	–	–	–
Mixed	63.0 (38.0–85.0)	97.1	15.5 (10.2–22.2)	–	29.0 (23.0–35.0)	98.6
Overall	18.0 (5.0–36.0)	99.4	18.0 (6.0–34.0)	98.7	71.0 (46.0–91.0)	99.7

– indicates statistics cannot be calculated, due either to no observation or only one study included

All the *p* values from the test of heterogeneity between groups are less than 0.001

**Fig. 2:**
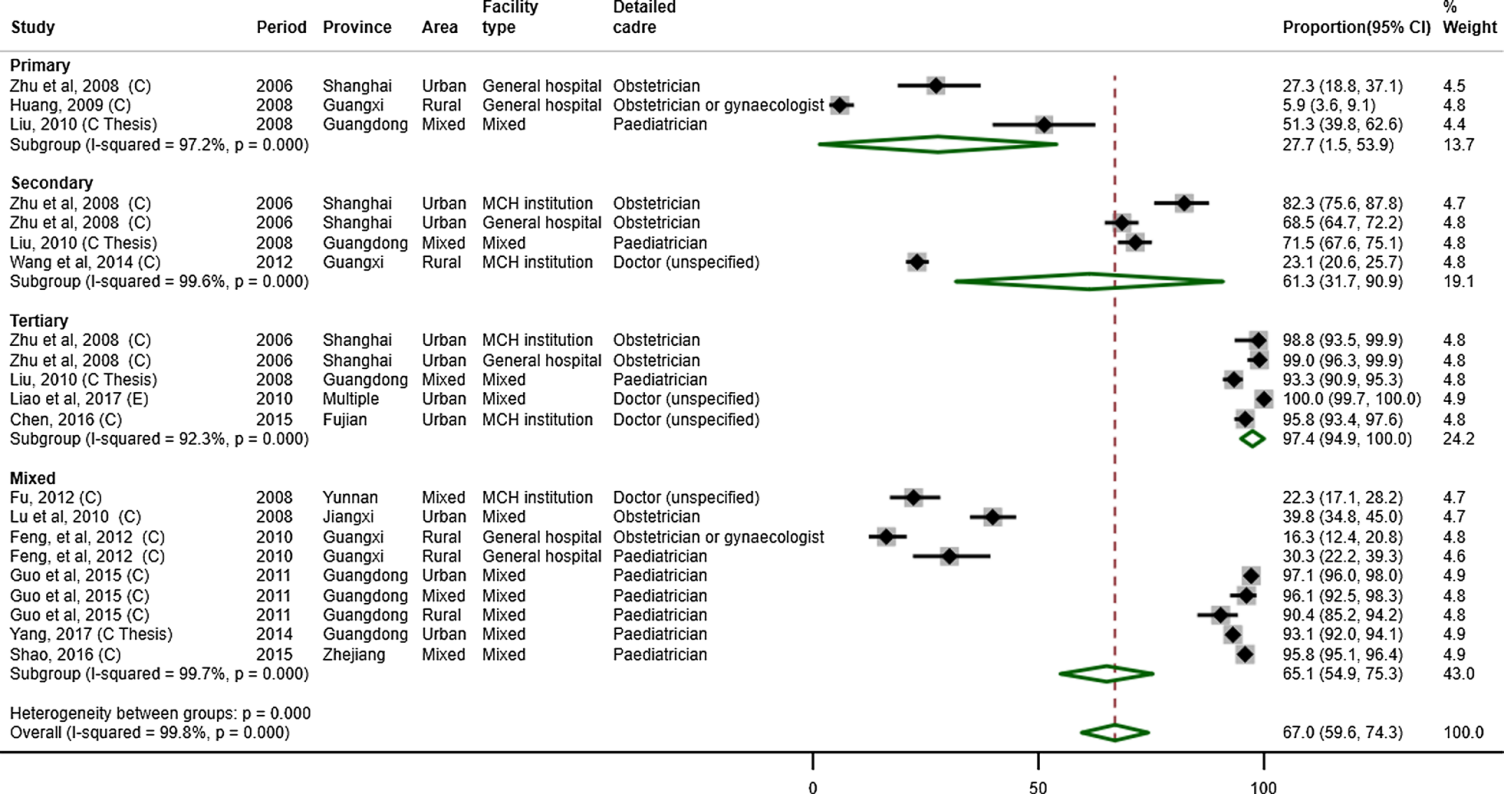
Forest plot showing the proportion of doctors holding bachelor or higher-level degrees

Subgroup meta-analyses stratified by facility level showed that lower-level health facilities had lower proportions of MCH workers with bachelor or higher-level degrees (Table 1). Subgroup meta-analyses lowered the I^2^ statistics for almost all the groups. For doctors, the pooled weighted average proportion of having bachelor or higher-level degrees was 27.7% (1.5–53.9%) at primary-level facilities (Fig. 2). The proportion for nurses was 1.0% (0.0–5.0%), while that for other cadres was 3.0% (0.0–12.0%) [see Additional file 4 for the full set of forest plots].

#### Health-related discipline

Twelve studies provided information on MCH workers’ health-related discipline (Table 2) [34, 46–50, 53, 55–59]. The one study reporting on paediatricians suggested that they had all studied clinical medicine whilst obstetricians and gynaecologist had degrees from either “Clinical medicine” or “Maternal and child health” (a subarea of “Public health”).

**Table 2:** Health-related discipline training received by MCH workers

Cadre (number of studies)	Health-related discipline
Obstetrician or gynaecologist (1)	Clinical medicine, Maternal and child healthcare
Paediatrician (1)	Clinical medicine
Nurse (1)	Nursing
Midwife (3)	Midwifery, Nursing, Clinical medicine
Specialized public health worker (3)	Clinical medicine, Nursing, Public health, Auxiliary medicine, Maternal and child healthcare
Vaccinator (2)	Clinical medicine, Traditional Chinese medicine, Nursing, Midwifery, Public health, Non-medical specialty
Health information worker (1)	Clinical medicine, Nursing, Health sciences, Computer sciences
Maternal health worker (1)	Nursing, Midwifery, Maternal and child healthcare, Clinical medicine

The total number of studies exceeds 12 because two studies provide information for different cadres of MCH workers

#### Certification

Only two studies provided information on MCH workers’ certification. One study in 2013 reported that only half (52.4%, 714 out of 1364) of the MCH workers from Chongqing held valid certification [62]. Among the other half of the MCH workers, 37.5% were incorrectly certificated not for the role in which they worked, and 10.1% provided MCH services without holding any certification. Similarly, another study surveyed all the obstetricians, obstetric nurses and midwives from township hospitals in Baise city, Guangxi, in 2008, finding that, respectively, 7.5% (24 out of 321 obstetricians), 68.5% (102 out of 149 obstetric nurses) and 4.7% (2 out of 43 midwives) of the MCH workers did not hold any certificate [33].

### Studies reporting the density of MCH workers

#### Study characteristics

The included studies are summarized by cadre [Additional file 5]. Of the 18 studies on density, 17 (94.4%) were done after 1990, and 5 (27.8%) were nationally representative [16, 63, 64, 72, 76]. The single-province studies included Zhejiang (*n* = 3), Anhui (*n* = 2), Guangdong (*n* = 2), Yunnan (*n* = 2), Shanghai (*n* = 1), Jiangsu (*n* = 1), Xinjiang (*n* = 1), and Liaoning (*n* = 1).

#### Density by cadre

**Fig. 3:**
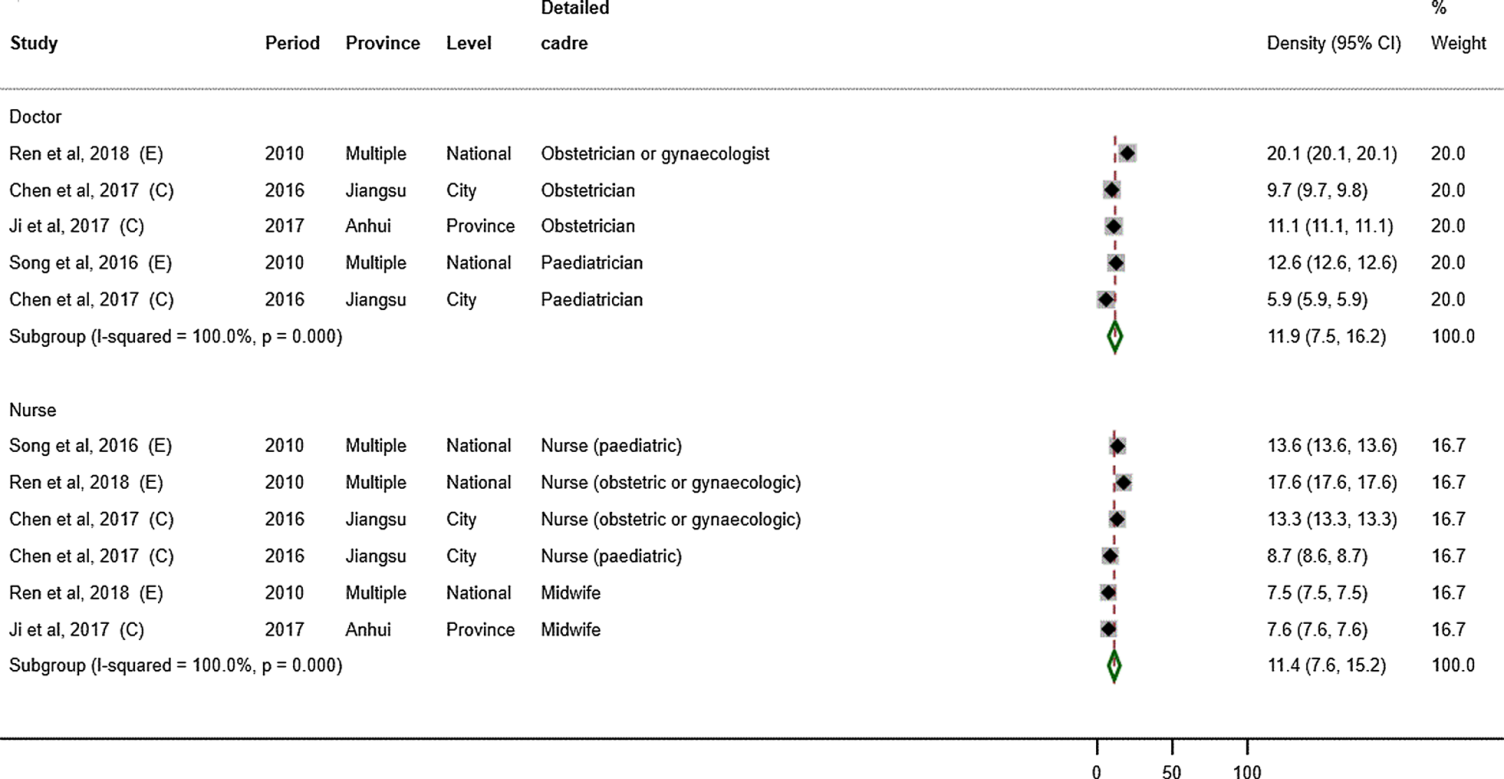
Forest plot showing the density of MCH workers by cadre, per 100 000 population

**Fig. 4:**
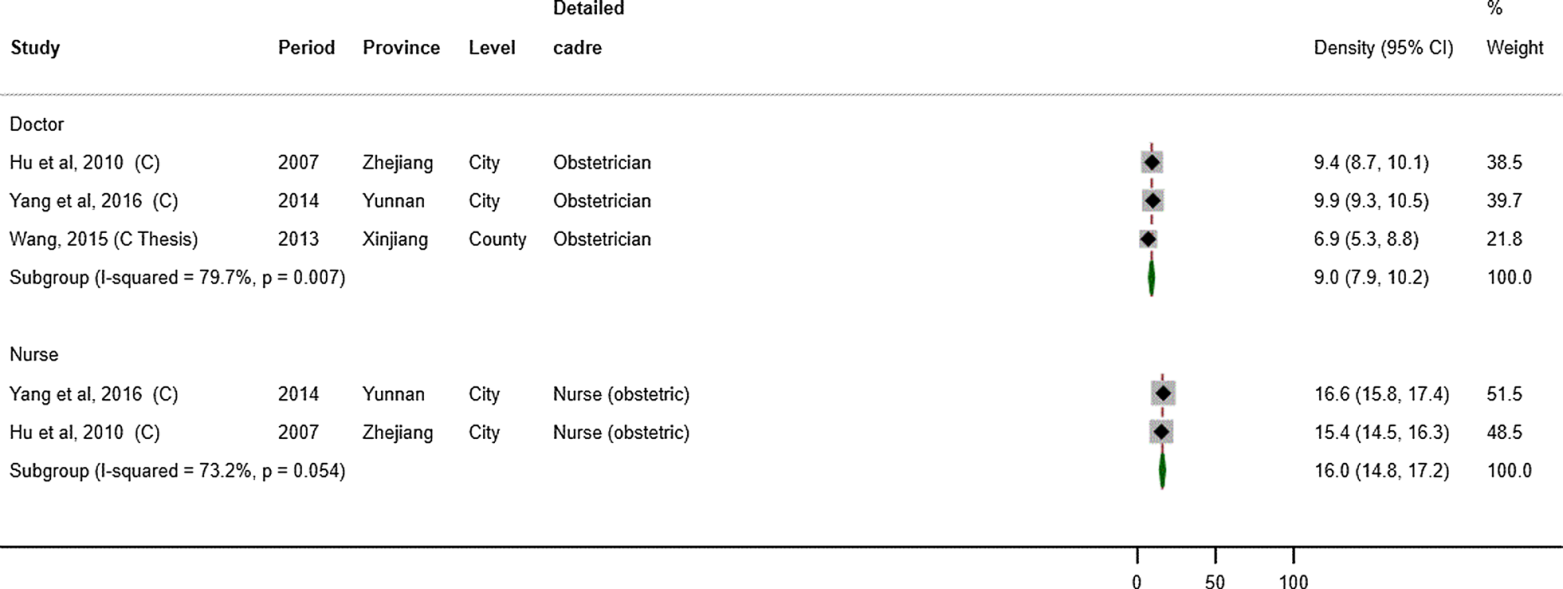
Forest plot showing the density of MCH workers by cadre, per 1000 births

For studies using total population as the denominator (Fig. 3), the weighted average density of MCH doctors was 11.9 (95% CI: 7.5–16.2) per 100 000 population (*n* = 5) and that of MCH nurses was 11.4 (7.6–15.2) (*n* = 6). For studies using number of births as the denominator (Fig. 4), the weighted average density of obstetricians was 9.0 (95% CI: 7.9–10.2) per 1000 births (*n* = 3) and that of obstetric nurses was 16.0 (14.8–17.2) per 1000 births (n = 2).

#### Ratios of MCH workers

**Fig. 5:**
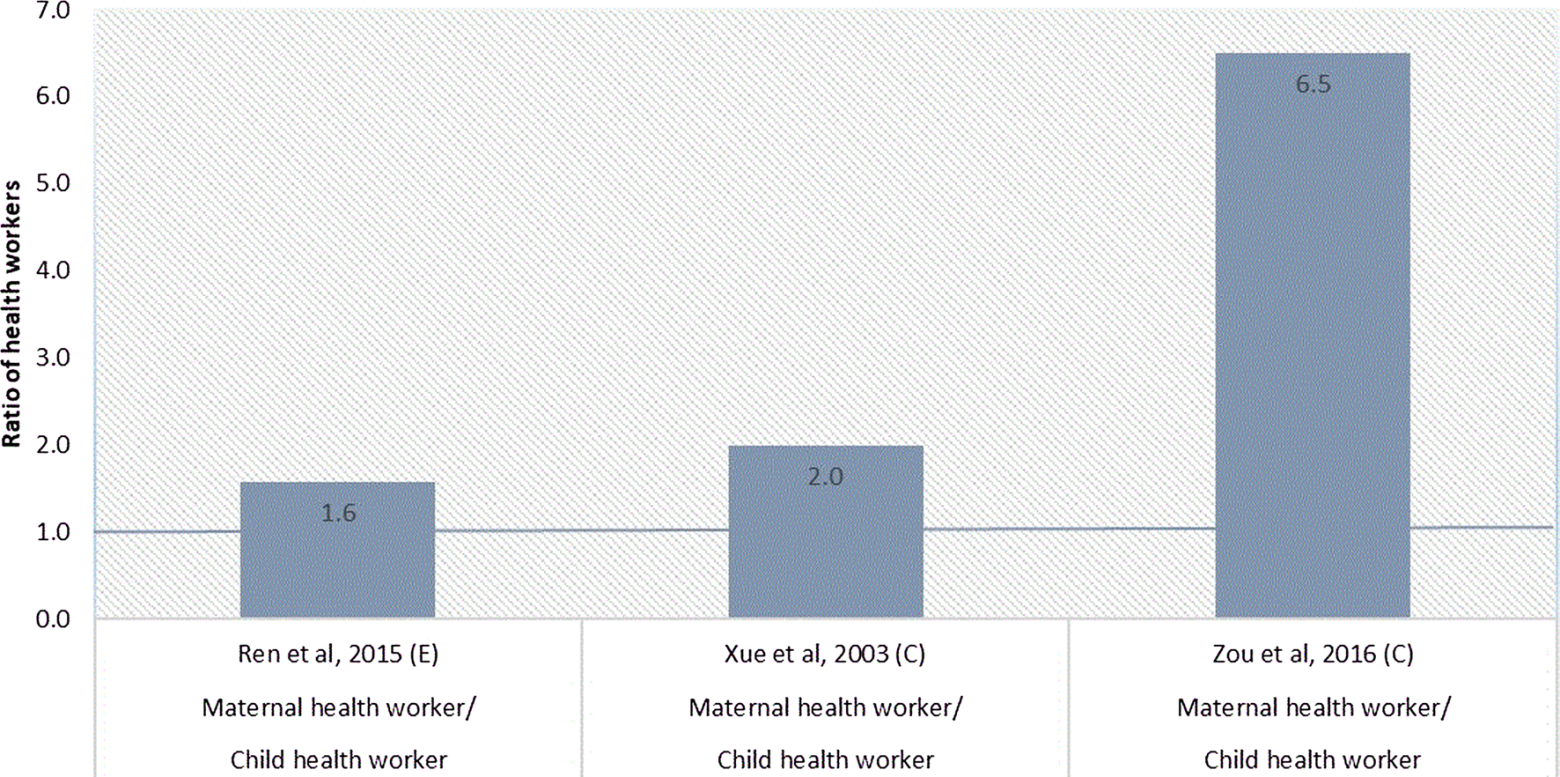
Maternal-to-child health worker ratio

**Fig. 6:**
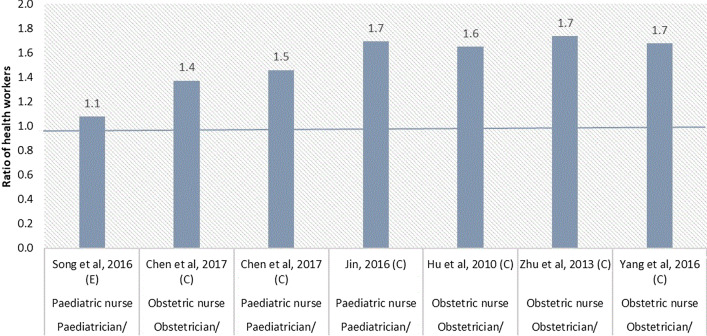
Nurse-to-doctor ratio

Three studies allowed us to calculate the ratio of maternal to child health workers (Fig. 5). The density of the maternal health workers was between 1.6 and 6.5 times higher than the density of child health workers. Six studies allowed us to calculate the ratio of MCH nurse density to MCH doctor density (Fig. 6). The ratio of obstetric nurses to obstetricians ranged from 1.4:1 to 1.7:1. The ratio of paediatric nurses to paediatricians ranged from 1.1:1 to 1.7:1.

## Discussion

The aim of the review was to describe the profile and density of MCH workers in China and to help understand how the MCH workforce development contributes to China’s achievement in MCH. The study has three main findings. First, only two-thirds of obstetricians and paediatricians had a bachelor or higher degree. This proportion was lower in primary-level facilities (28%). For nurses involved in MCH care the proportions with a bachelor or higher degree were lower (20.0% in any health facility and 1% in primary care facilities). Second, the proportions of MCH workers who held a certificate—a rigorous system introduced by the Chinese Government to regulate who can perform MCH tasks—ranged from 32% (47 of 149 obstetric nurses) to 95% (41 out of 43 midwives) in primary care facilities. Third, the average density of obstetricians was 9.0 (7.9–10.2) per 1000 births and that of obstetric nurses 16.0 (14.8–17.2) per 1000 births. The density of MCH workers is much higher than what has been recommended internationally (three doctors and 20 midwives per 3600 births).

Obstetricians, paediatricians and other MCH worker cadres were much less educated at the primary level than at the tertiary level. That is partly because most medical school graduates in China compete to join large hospitals, where their salaries, working conditions and career opportunities are superior to those offered by primary-level health facilities [78]. The mobility rate of experienced and qualified health workers in primary-level health facilities is high [79]. The lower capacity of MCH workers at the primary level is also seen in other low- and -middle-income countries (LMICs). China’s strategy has been to achieve widespread deployment of the workforce first and then to upgrade the skills of the providers to improve the standard of care gradually. Such strategy contributed to the higher density of obstetricians and obstetric nurses as compared to the international benchmark. Similar strategies are also seen in the expansion of the insurance schemes, where universal health insurance coverage is achieved first and an improvement of the benefit package follows [80].

Although the education level of MCH workers is variable, China achieved near-universal access to childbirth in health facilities with low maternal mortality and neonatal mortality as a result [3, 81]. Childbirth is now concentrated in well-staffed hospitals at secondary and tertiary levels, whereas primary-level facilities focus on antenatal care and screening of high-risk pregnancies [3]. Concentrating births in large facilities facilitates an efficient and effective midwifery and obstetric skill mix, with providers being highly trained and equipped to ensure safe birth. The government no longer allows caesarean sections to be provided at township hospitals, where maternal health workers now focus on home visits, birth preparedness, and postpartum follow-up. In addition, accredited obstetricians from tertiary facilities regularly visit secondary or primary-level facilities to provide in-service training and supervision [3]. Evidence from other countries has shown that some MCH tasks do not require advanced skills and health workers with no advanced training can perform well provided that they get supervision from higher-level facilities [82]. Another example is immunization, which is not a complex intervention. Although primary-level facilities are poorly staffed for highly medicalized services, immunization is offered largely at primary care level. In rural areas, immunization is actively promoted by village doctors, who only have basic levels of education.

Certification of MCH workers is an important strategy to ensure that particular tasks are only performed by those skilled and equipped to do so [83, 84]. We found that not all MCH workers held a valid certificate. The finding does not necessarily imply that this is a failure of certification mechanism. Health facilities are required to comply with the regulations relating to certification of MCH workers. But there is a lack of information on the degree of compliance of health facilities with the regulations. It could be partly due to the fact that limited resources have been put in place to enforce the certification law. Although rare evidence exists, this problem might also occur in private hospitals, which are often less regulated.

Our study has found that for density of MCH workers, the population denominator has been used more often than birth denominator. The weighted average density of MCH doctors reported in our study (11.9 per 100 000 population) was similar to that in Sweden and France in 2012—12.1 and 12.3, respectively [85]. However, fertility in these countries is higher than in China, so comparisons may not be valid. We echo Gabrysch and colleagues’ recommendations about the need to enhance discriminatory power of density indicators and measure density of different cadres of MCH workers according to specific demographic profile, for example, define density of obstetricians using per births instead of per population [20].

Through the analysis of MCH worker ratio, we found a larger maternal health workforce than child health workforce, and more nurses than doctors. Although no gold standard exists for the ratio of maternal to child health workers, the shortage of child health workers in China has been a long concern. Paediatrics is not a popular choice for medical students due to the heavy workload, low salary compared with other medical professions and intense doctor–patient relationships [86]. The revealed deficit in the availability of child health workers needs to be addressed through implementing targeted human resource policies, such as through the salary and bonus systems or improved working conditions. Compared with the optimal 2:1 nurse-to-doctor ratio as recommended by WHO [19], the nurse-to-doctor ratio in MCH area was less than optimal. China has a long history of having a low nurse-to-doctor ratio. The nurse-to-doctor ratio was estimated around 1:10 in the early 1950s (WHO, 2015) [87]. The situation was reversed with an estimated nurse-to-doctor ratio of 1.1:1 in 2019 [88]. China still needs to step up its training of nurses to perform the MCH services.

In the era of SDGs, many countries address MCH workforce challenges through task-shifting and creating cadres capable of providing antenatal care, intrapartum care, postpartum care and paediatric care [89]. It is necessary to look at the MCH workforce as a whole in-country systems to complement to the measurement of particular cadres of MCH workers. While the presented MCH workforce analysis was confined to mainland China, the analysis we have done is likely to apply to many LMICs which do not apply international training standards, e.g. Vietnam, Myanmar and Zambia [90]. As countries try to address MCH workforce gaps, reliable and up-to-date information on the profile and density of MCH workers is urgently needed for evidence-based policy making. This calls for improved methods in future primary data collection, including clear definitions of MCH workers and robust measurement of MCH workforce density.

Our study has several limitations. First, we found no studies reporting on the private sectors, where the profile of MCH workers may be different from those working in the public sector. Second, we only focused on the length of education without analysing the content of training, so we did not assess the skills of the MCH workers. Third, we did not separate the data by year for the meta-analysis because that would result in too few studies for each cadre and each subgroup. Given that 94% of the included studies were done after 1990 and health workforce usually takes a decade or a generation to develop [91], the difference in time period was unlikely to change the results. Fourth, there were relatively too few studies contributing to each subgroup (less than 10 studies). Subgroup proportions need to be interpreted with uncertainty. Fifth, there could be some mis-reporting given the methods used to report on MCH workers. Last, the quality of the included studies needs careful scrutiny, because there is unclear and high risk of bias in almost all studies.

## Conclusion

The high density of MCH workers in China is achieved through a mix of workers with high and low educational profiles. Many workers labelled as “obstetricians” or “paediatrician” have lower qualifications than expected. China compensates for these low educational levels through task-shifting, in-service training and supervision. In a global context, particularly in the area of maternal and newborn health, many countries are pushing for degree-level qualification of skilled health professionals. China’s experience in training and optimizing the roles of less educated MCH workers can be further explored as a strategic option for poor-resourced settings.

## Supplementary Information

**Additional file 1. **Search strategy for English and Chinese literature on China’s MCH workforce.

**Additional file 2. **Quality assessment of included studies.

**Additional file 3. **Studies reporting on MCH workforce profile: study design and density.

**Additional file 4. **Meta-analysis for proportions of MCH workers with different education levels.

**Additional file 5. **Studies reporting on MCH workforce density: study design and density.

## Data Availability

Data are available from the corresponding author upon request.
