# Supplementary material for: A systematic review of the profile and density of the maternal and child health workforce in China
Source: Hum Resour Health. 2021 Oct 9;19:125. doi: 10.1186/s12960-021-00662-4 (PMC8501553; doi:10.1186/s12960-021-00662-4)
Supplement: Supplementary file 1 — Additional file 1. Search strategy for English and Chinese literature on China’s MCH workforce. [file 12960_2021_662_MOESM1_ESM.docx]

**Additional file 1**

**A1. Search strategy for English and Chinese literature on China’s MCH workforce**

**A1.1 English database search**

Embase: 1517

Medline: 941

Cochrane: 71

Econlit: 5

Web of Science: 1357

Global Health: 1108

6 databases in total: 4999

Duplicated references：delete 1429 duplicates

Final dataset: 3570

**Embase Classic+Embase@Ovid**

| No. | Classification | Term | Results |
| --- | --- | --- | --- |
| 1 | Human resources for health | exp medical profession/ or exp paramedical personnel/ or exp medical education/ or exp medical student/ or exp Medical Staff/ or exp Health Care Manpower/ or exp Health Personnel/ or exp health practitioner/ or exp residency education/ or exp health student/ or exp medical personnel/ or 'health worker*'.tw. or 'health workforce*'.tw. or 'health personnel'.tw. or HRH.tw. or HHR.tw. or 'health human resource*'.tw. or 'medical staff'.tw. or 'health*care provider*'.tw. or 'health*care worker*'.tw. or 'health professional*'.tw. or 'health*care professional*'.tw. or 'health*care workforce*'.tw. or 'health provider*'.tw. or 'medical staff'.tw. or 'medical workforce*'.tw. or 'health*care personnel'.tw. or 'health personnel'.tw. 'Health*care Practitioner*'.tw. or 'health practitioner*'.tw. or 'medical student*'.tw. or 'med student*'.tw. or 'health student*'.tw. or 'medical graduate*'.tw. or physician*.tw. or doctor*.tw. or surgeon*.tw. or internist*.tw.or 'general practitioner*'.tw. or nurse*.tw. or 'skilled birth attendant*'.tw. or 'family planning personnel'.tw. or 'medical technician*'.tw. or 'midwifery workforce*'.tw. or midwi?e*.tw. or 'nursing staff'.tw. or 'nursing personnel'.tw. or 'nursing workforce*'.tw. or 'nursing professional*'.tw. or pharmacist.tw. or pharmacists.tw. or 'health care practitioner*'.tw. or 'health care personnel'.tw. or 'health care workforce*'.tw. or 'health care professional*'.tw. or 'health care worker*'.tw. or 'health care provider*'.tw. or paediatrician*.tw. or pediatrician*.tw. or obstetrist*.tw. or obstetrician*.tw. or tocologist*.tw. or gynaecologist*.tw. or gynecologist*.tw. or (human resource* adj4 health).tw. or 'obstetric* workforce*'.tw. or 'paediatric* workforce*'.tw. or 'pediatric* workforce*'.tw. or 'vaccination staff'.tw. or vaccinator*.tw. or 'vaccination personnel'.tw. or 'obstetric* personnel'.tw. or 'paediatric* personnel'.tw. or 'paediatric* personnel'.tw. | 2256760 |
| 2 | MCH services | exp Child Health Services/ or exp Maternal Health Services/ or exp Cesarean Section/ or exp Maternal-Child Nursing/ or exp Obstetric Nursing/ or exp Prenatal Care/ or exp Prenatal Diagnosis/ or exp Home Childbirth/ exp Pediatric Nursing/ or exp Maternal Health Services/ or exp Maternal Health/ or exp Child Care/ or exp Child Health Services/ or exp Intensive Care, Neonatal/ or exp Child Health/ or exp Vaccination/ or exp Immunization/ or exp BCG Vaccine/ or exp Measles Vaccine/ or exp Diphtheria-Tetanus-Pertussis Vaccine/ exp Hepatitis B Vaccines/ or maternal health*.tw. or child health*.tw. or neonat* health*.tw. or MCH.tw. or MNH.tw. or MDG4.tw. or MDG5.tw. or millennium development goal*.tw. or safe motherhood.tw. or prenatal.tw. or pre-natal.tw. or antenatal.tw. or ante-natal.tw. or postnatal.tw. or post-natal.tw. or c-section*.tw. or caesarean section*.tw. or cesarean section*.tw. or facility deliver*.tw. or facility-based deliver*.tw. or home birth.tw. or homebirth.tw. or child birth.tw. or childbirth.tw. or home deliver*.tw. or skilled deliver*.tw. or institutional* deliver*.tw. or vaccin*.tw. or immuniz*.tw. or immunis*.tw. or EPI.tw. | 1272246 |
| 3 | China | exp China/ or China.tw. or Chinese.tw. | 391966 |
| 4 |  | #1 AND #2 AND #3 | 1517 |

**Medline@Ovid**

| No. | Classification | Term | Results |
| --- | --- | --- | --- |
| 1 | Human resources for health | exp medical profession/ or exp paramedical personnel/ or exp medical education/ or exp medical student/ or exp Medical Staff/ or exp Health Manpower/ or exp Health Personnel/ or exp Nurse Practitioners/ exp Internship and Residency/ or 'health worker*'.tw. or 'health workforce*'.tw. or 'health personnel'.tw. or HRH.tw. or HHR.tw. or 'health human resource*'.tw. or 'medical staff'.tw. or 'health*care provider*'.tw. or 'health*care worker*'.tw. or 'health professional*'.tw. or 'health*care professional*'.tw. or 'health*care workforce*'.tw. or 'health provider*'.tw. or 'medical staff'.tw. or 'medical workforce*'.tw. or 'health*care personnel'.tw. or 'health personnel'.tw. or 'Health*care Practitioner*'.tw. or 'health practitioner*'.tw. or 'medical student*'.tw. or 'med student*'.tw. or 'health student*'.tw. or 'medical graduate*'.tw. or Physician*.tw. or doctor*.tw. or surgeon*.tw. or or internist*.tw. or general practitioner*.tw. or nurse*.tw. or 'skilled birth attendant*'.tw. or 'family planning personnel'.tw. or 'medical technician*'.tw. or 'midwifery workforce*'.tw. or midwi?e*.tw. or 'nursing staff'.tw. or 'nursing personnel'.tw. or 'nursing workforce*'.tw. or 'nursing professional*'.tw. or pharmacist.tw. or pharmacists.tw. or 'health care practitioner*'.tw. or 'health care personnel'.tw. or 'health care workforce*'.tw. or 'health care professional*'.tw. or 'health care worker*'.tw. or 'health care provider*'.tw. or paediatrician*.tw. or pediatrician*.tw. or obstetrist*.tw. or obstetrician*.tw. or tocologist*.tw. or gynaecologist*.tw. or gynecologist*.tw. or (human resource* adj4 health).tw. or 'obstetric* workforce*'.tw. or 'paediatric* workforce*'.tw. or 'pediatric* workforce*'.tw. or 'vaccination staff'.tw. or vaccinator*.tw. or 'vaccination personnel'.tw. or 'obstetric* personnel'.tw. or 'paediatric* personnel'.tw. or 'paediatric* personnel'.tw. | 1328681 |
| 2 | MCH services | exp Child Health Services/ or exp Maternal Health Services/ or exp Cesarean Section/ or exp Maternal-Child Nursing/ or exp Obstetric Nursing/ or exp Prenatal Care/ or exp Prenatal Diagnosis/ or exp Home Childbirth/ or exp Obstetric Nursing/ or exp Pediatric Nursing/ or exp Maternal Health/ or exp Child Care/ or exp Child Health Services/ or exp Intensive Care, Neonatal/ or exp Child Health/ or exp Vaccination/ or exp Immunization/ or exp BCG Vaccine/ or exp Measles Vaccine/ or exp Diphtheria-Tetanus-Pertussis Vaccine/ or exp Hepatitis B Vaccines/ or 'maternal health*'.tw. or 'child health*'.tw. or 'neonat* health*'.tw. or MCH.tw. or MNH.tw. or MDG4.tw. or MDG5.tw. or 'millennium development goal*'.tw. or 'safe motherhood'.tw. or prenatal.tw. or pre-natal.tw. or antenatal.tw. or ante-natal.tw. or postnatal.tw. or post-natal.tw. or c-section*.tw.  or 'caesarean section*'.tw. or 'cesarean section*'.tw. or 'facility deliver*'.tw. or 'facility-based deliver*'.tw. or 'home birth'.tw. or homebirth.tw. or 'child birth'.tw. or childbirth.tw. or 'home deliver*'.tw. or 'skilled deliver*'.tw. or 'institutional* deliver*'.tw. or EPI.tw. or vaccin*.tw. or immuniz*.tw. or immunis*.tw. | 850849 |
| 3 | China | exp China/ or China.tw. or Chinese.tw. | 285572 |
| 4 |  | #1 AND #2 AND #3 | 941 |

**Cochrane Library**

| No. | Classification | Term | Results |
| --- | --- | --- | --- |
| 1 | Human resources for health | MeSH descriptor: [Health Manpower] explode all trees or MeSH descriptor: [Medical Staff] explode all trees or MeSH descriptor: [Allied Health Personnel] explode all trees or MeSH descriptor: [Health Personnel] explode all trees or MeSH descriptor: [Community Health Workers] explode all trees or MeSH descriptor: [Allied Health Occupations] explode all trees or MeSH descriptor: [Education, Medical] explode all trees or MeSH descriptor: [Students, Medical] explode all trees or ‘health worker*’:ti,ab,kw or ‘health workforce*’:ti,ab,kw or  ‘health personnel’:ti,ab,kw or HRH:ti,ab,kw or HHR:ti,ab,kw or ‘health human resource*’:ti,ab,kw or ‘medical staff’:ti,ab,kw or ‘health*care provider*’:ti,ab,kw or ‘health care provider*’:ti,ab,kw or ‘health care worker*’:ti,ab,kw or ‘health*care worker*’:ti,ab,kw or ‘health professional*’:ti,ab,kw or ‘health care professional*’:ti,ab,kw or ‘health*care professional*’:ti,ab,kw or ‘health*care workforce*’:ti,ab,kw or ‘health care workforce*’:ti,ab,kw or ‘health provider*’:ti,ab,kw or ‘health practitioner*’:ti,ab,kw or ‘medical workforce*’:ti,ab,kw or ‘medical personnel’:ti,ab,kw or ‘medical practitioner*’:ti,ab,kw or ‘health care personnel’:ti,ab,kw or ‘health*care personnel’:ti,ab,kw or ‘health care practitioner*’:ti,ab,kw or ‘health*care practitioner*’:ti,ab,kw or ‘human resource*’ near/4 health or ‘medical student*’:ti,ab,kw or ‘med student*’:ti,ab,kw or ‘health student*’:ti,ab,kw or ‘medical graduate*’:ti,ab,kw or physician*:ti,ab,kw or  doctor*:ti,ab,kw or surgeon*:ti,ab,kw or internist*:ti,ab,kw or ‘general practitioner*’:ti,ab,kw or nurse*:ti,ab,kw or ‘skilled birth attendant*’:ti,ab,kw or ‘family planning personnel’:ti,ab,kw or ‘midwifery workforce’:ti,ab,kw or midwi?e*:ti,ab,kw or ‘nursing staff’:ti,ab,kw or ‘nursing personnel’:ti,ab,kw or  ‘nursing workforce’:ti,ab,kw or ‘nursing professional*’:ti,ab,kw or dentist*:ti,ab,kw or pharmacist*:ti,ab,kw or  ‘medical technician*’:ti,ab,kw | 75398 |
| 2 | MCH services | MeSH descriptor: [Maternal Health] explode all trees or MeSH descriptor: [Maternal Health Services] explode all trees or MeSH descriptor: [Child Health] explode all trees or MeSH descriptor: [Intensive Care, Neonatal] explode all trees or MeSH descriptor: [Child Health Services] explode all trees or MeSH descriptor: [Infant Health] explode all trees or MeSH descriptor: [Perinatal Care] explode all trees or MeSH descriptor: [Neonatal Nursing] explode all trees or MeSH descriptor: [Prenatal Care] explode all trees or MeSH descriptor: [Postnatal Care] explode all trees or MeSH descriptor: [Cesarean Section] explode all trees or MeSH descriptor: [Delivery, Obstetric] explode all trees or MeSH descriptor: [Parturition] explode all trees or MeSH descriptor: [Pregnancy] explode all trees or MeSH descriptor: [Immunization] explode all trees or MeSH descriptor: [Vaccination] explode all trees or ‘maternal health*’:ti,ab,kw or ‘child health*’:ti,ab,kw or ‘neonat* health*’:ti,ab,kw or MCH:ti,ab,kw or MNH:ti,ab,kw or MDG4:ti,ab,kw or MDG5:ti,ab,kw or millennium development goal*:ti,ab,kw or safe motherhood:ti,ab,kw or prenatal:ti,ab,kw or pre-natal:ti,ab,kw or antenatal:ti,ab,kw or ante-natal:ti,ab,kw or postnatal:ti,ab,kw or post-natal:ti,ab,kw or c-section*:ti,ab,kw or  caesarean section*:ti,ab,kw or cesarean section*:ti,ab,kw or facility deliver*:ti,ab,kw or facility-based deliver*:ti,ab,kw or ‘home birth’:ti,ab,kw or homebirth:ti,ab,kw or ‘child birth’:ti,ab,kw or childbirth:ti,ab,kw or ‘home deliver*’:ti,ab,kw or ‘skilled deliver*’:ti,ab,kw or ‘institutional* deliver*’:ti,ab,kw or vaccin*:ti,ab,kw or immuniz*:ti,ab,kw or immunis*:ti,ab,kw or EPI:ti,ab,kw or | 42671 |
| 3 | China | MeSH descriptor: [China] explode all trees or ‘china’:ti,ab,kw or ‘Chinese’:ti,ab,kw | 22427 |
| 4 |  | #1 AND #2 AND #3 | 71 |

**Econlit@Ovid**

| No. | Classification | Term | Results |
| --- | --- | --- | --- |
| 1 | Human resources for health | 'health worker*'.tw. or 'health workforce*'.tw. or 'health personnel'.tw. or HRH.tw. or HHR.tw. or 'health human resource*'.tw. or 'medical staff'.tw. or 'health$care provider$'.tw. or 'health$care worker$'.tw. or 'health professional*'.tw. or 'health$care professional*'.tw. or 'health$care workforce*'.tw. or 'health provider$'.tw. or 'medical staff'.tw. or 'medical workforce*'.tw. or 'health$care personnel'.tw. or 'health personnel'.tw. or 'Health$care or practitioner*'.tw. or 'health practitioner*'.tw. or 'medical student*'.tw. or 'med student*'.tw. or 'health student*'.tw. or 'medical graduate*'.tw. or Physician*.tw. or doctor*.tw. or surgeon*.tw. or internist*.tw. or general practitioner*.tw. or nurse*.tw. or 'skilled birth attendant*'.tw. or 'family planning personnel'.tw. or 'medical technician*'.tw. or 'midwifery workforce*'.tw. or midwi?e*.tw. or 'nursing staff'.tw. or 'nursing personnel'.tw. or 'nursing workforce*'.tw. or 'nursing professional*'.tw. or pharmacist.tw. or pharmacists.tw. or ‘health care practitioner*’.tw. or ‘health care personnel’.tw. or ‘health care workforce*’.tw. or ‘health care professional*’.tw. or ‘health care worker*’.tw. or ‘health care provider*’.tw. or paediatrician*.tw. or pediatrician*.tw. or obstetrist*.tw. or obstetrician*.tw. or tocologist*.tw. or gynaecologist*.tw. or gynecologist*.tw. or (human resource* adj4 health).tw. or ‘obstetric* workforce*’.tw. or ‘paediatric* workforce*’.tw. or ‘pediatric* workforce*’.tw. or resident*.tw. or ‘vaccination staff’.tw. or vaccinator*.tw. or  ‘vaccination personnel’.tw. or ‘obstetric* personnel’.tw. or ‘paediatric* personnel’.tw. or ‘paediatric* personnel’.tw. | 7142 |
| 2 | MCH services | maternal health*.tw. or child health*.tw. or neonat* health*.tw. or MCH.tw. or MNH.tw. or MDG4.tw. or MDG5.tw. or millennium development goal*.tw. or safe motherhood.tw. or prenatal.tw. or pre-natal.tw. or antenatal.tw. or ante-natal.tw. or postnatal.tw. or post-natal.tw. or c-section*.tw. or caesarean section*.tw. or cesarean section*.tw. or facility deliver*.tw. or facility-based deliver*.tw. or home birth.tw. or homebirth.tw. or child birth.tw. | 3777 |
| 3 | China | China.tw. or Chinese.tw. | 38851 |
| 4 |  | #1 AND #2 AND #3 | 5 |

**Web of Science**

| No. | Classification | Term | Results |
| --- | --- | --- | --- |
| 1 | Human resources for health | ts=(‘medical professional*’ or ‘medical personnel’ or ‘health personnel’ or ‘health human resource*’ or ‘health workforce*’ or ‘medical staff’ or ‘healthcare provider*’ or ‘health-care provider*’ or ‘health care worker*’ or surgeon* or internist* or ‘healthcare worker*’ or ‘health professional*’ or ‘health care professional*’ or ‘health worker*’ or practitioner* or physician* or doctor* or nurse* or pharmacist* or physician* or ‘skilled birth attendant*’ or midwife* or ‘midwifery workforce*’ or midwives or ‘health manpower’ or HRH or HHR or paediatrician* or pediatrician* or obstetrist* or obstetrician* or tocologist* or gynaecologist* or gynecologist* or ‘human resource* adj4 health’ or ‘obstetric* workforce*’ or ‘paediatric* workforce*’ or ‘pediatric* workforce*’ or ‘vaccination staff’ or vaccinator* or ‘vaccination personnel’ or ‘obstetric* personnel’ or ‘paediatric* personnel’ or ‘pediatric* personnel’) | 887960 |
| 2 | MCH services | ts=(maternal health* or child health* or neonat* health* or infant health* or MCH or MNH or MDG4 or MDG5 or millennium development goal* or safe motherhood or prenatal or pre-natal or antenatal or ante-natal or postnatal or post-natal or c-section* or caesarean section* or cesarean section* or facility deliver* or facility-based deliver* or hospital* deliver* or hospital-based deliver* or home birth or home birth or home deliver* or skilled deliver* or institutional* deliver* or vaccin* or immuniz* or immunis* or EPI) | 928149 |
| 3 | China | ts=(China or Chinese) | 699607 |
| 4 |  | #1 AND #2 AND #3 | 1357 |

**Global Health**

| No. | Classification | Term | Results |
| --- | --- | --- | --- |
| 1 | Human resources for health | exp health care workers/ or exp medical education/ or exp medical student/ or 'health worker*'.tw. or 'health workforce*'.tw. or 'health personnel'.tw. or HRH.tw. or HHR.tw. or 'health human resource*'.tw. or 'medical staff'.tw. or 'health$care provider$'.tw. or 'health$care worker$'.tw. or 'health professional*'.tw. or 'health$care professional*'.tw. or 'health$care workforce*'.tw. or 'health provider$'.tw. or 'medical staff'.tw. or 'medical workforce*'.tw. or 'health$care personnel'.tw. or 'health personnel'.tw. or 'Health$care Practitioner*'.tw. or 'health practitioner*'.tw. or 'medical student*'.tw. or 'med student*'.tw. or 'health student*'.tw. or 'medical graduate*'.tw. or Physician*.tw. or doctor*.tw. or surgeon*.tw. or internist*.tw. or general practitioner*.tw. or nurse*.tw. or 'skilled birth attendant*'.tw. or 'family planning personnel'.tw. or 'medical technician*'.tw. or 'midwifery workforce*'.tw. or midwi?e*.tw. or 'nursing staff'.tw. or 'nursing personnel'.tw. or 'nursing workforce*'.tw. or 'nursing professional*'.tw. or pharmacist.tw. or pharmacists.tw. or ‘health care practitioner*’.tw. or ‘health care personnel’.tw. or ‘health care workforce*’.tw. or ‘health care professional*’.tw. or ‘health care worker*’.tw. or ‘health care provider*’.tw. or paediatrician*.tw. or pediatrician*.tw. or obstetrist*.tw. or obstetrician*.tw. or tocologist*.tw. or gynaecologist*.tw. or gynecologist*.tw. or (human resource* adj4 health).tw. or ‘obstetric* workforce*’.tw. or ‘paediatric* workforce*’.tw. or ‘pediatric* workforce*’.tw. or resident*.tw. or ‘vaccination staff’.tw. or vaccinator*.tw. or ‘vaccination personnel’.tw. or ‘obstetric* personnel’.tw. or ‘paediatric* personnel’.tw. or ‘paediatric* personnel’.tw. | 187811 |
| 2 | MCH services | exp Prenatal Care/ or exp Prenatal Education/ or exp Prenatal Diagnosis/ or exp Child Care/ or exp Child Health/ or exp Vaccination/ or exp Immunization/ or exp BCG Vaccine/ or maternal health*.tw. or child health*.tw. or neonat* health*.tw. or MCH.tw. or MNH.tw. or MDG4.tw. or MDG5.tw. or millennium development goal*.tw. or safe motherhood.tw. or prenatal.tw. or pre-natal.tw. or antenatal.tw. or ante-natal.tw. or postnatal.tw. or post-natal.tw. or c-section*.tw. or caesarean section*.tw. or cesarean section*.tw. or facility deliver*.tw. or facility-based deliver*.tw. or home birth.tw. or homebirth.tw. or child birth.tw. or childbirth.tw.  home deliver*.tw. or skilled deliver*.tw. or institutional* deliver*.tw. or vaccin*.tw. or immuniz*.tw. or immunis*.tw. or EPI.tw. | 221331 |
| 3 | China | exp China/ or China.tw. or Chinese.tw. | 194448 |
| 4 |  | #1 AND #2 AND #3 | 1108 |

**A1.2 Chinese database search**

CNKI: 20792

Wanfang: 6464

Two databases in total: 27256

Duplicated references：delete 927 duplicates

Final Chinese database: 26329

**China National Knowledge Infrastructure (CNKI)**

| No. | Classification | Searching terms | Results |
| --- | --- | --- | --- |
| 1 | Human resources for health | SU=卫生人力+卫生人才+卫生人员+医学人力+医学人才+医院人力+医务人员+医务工作者+卫生服务者+护理人力+护理人才+护理人员+药学人力+药学人才+药剂师+医技人才+医技人员+助产士+产科人力+产科人才+产科专家+儿科人力+儿科人才+儿科专家+接种人员+疫苗接种者+接生人员+接生人力+接生者+接生婆+医生+护士+大夫  Translation:  SU= health manpower + health talents + health staff + medical manpower + medical talents + hospital staff+ medical staff+ medical workers+ nursing workforce + nursing talents + nursing staff + pharmaceutical talents + pharmaceutical workforce +[pharmacist](file:///C:\Users\lsh410471\AppData\Local\youdao\dict\Application\7.5.0.0\resultui\dict\?keyword=pharmacist)s + technical talents + technical talents + midwives +[obstetric](file:///C:\Users\lsh410471\AppData\Local\youdao\dict\Application\7.5.0.0\resultui\dict\?keyword=obstetrics) manpower +[obstetric](file:///C:\Users\lsh410471\AppData\Local\youdao\dict\Application\7.5.0.0\resultui\dict\?keyword=obstetrics) talents + obstetric professionals+ paediatric personnel + paediatric talents + paediatric professionals + vaccination personnel + vaccination staff + midwifery personnel + midwifery workforce +midwifery workers + howdie + doctor + nurse + physician | 606345 |
| 2 | MCH services | SU=妇幼保健+妇幼服务+妇幼健康+妇幼卫生+孕期卫生服务+孕产期保健+孕产妇+围产期保健+围生保健+剖腹产+剖宫产+分娩方式+产科护理+住院分娩+孕期保健+孕前保健+产前保健+孕前护理+产前护理+产前检查+儿童健康+儿科护理+儿童保健+婴儿卫生服务+新生儿保健+新生儿护理+新生儿健康+接种+疫苗+计划免疫+免疫规划  Translation:  SU= maternal and child service + maternal and child healthcare + maternal and child health + maternal and child hygiene + pregnancy health care + [perinatal](file:///C:\Users\lsh410471\AppData\Local\youdao\dict\Application\7.5.0.0\resultui\dict\?keyword=perinatal)health [care](file:///C:\Users\lsh410471\AppData\Local\youdao\dict\Application\7.5.0.0\resultui\dict\?keyword=care) + pregnant women+ perinatal health care+ perinatal care+ caesarean section+ cesarean section+ delivery + obstetric care+ hospital delivery+ pregnancy care+ pre-pregnancy health care+ prenatal health care+ pre-pregnancy care+ prenatal care+ prenatal test+ health care card+ child health+ pediatric care+ child health care+ infant health care+ neonatal care + neonatal health + vaccination+ vaccine+ planned immunization + immunization programme | 450129 |
| 3 |  | 1 AND 2 | 20792 |

SU means searching for title, keyword and abstract.

**Wanfang database**

| No. | Classification | Searching terms | Results |
| --- | --- | --- | --- |
| 1 | Human resources for health | 题名或关键词:(卫生人力+卫生人才+卫生人员+医学人力+医学人才+医院人力+医务人员+医务工作者+卫生服务者+护理人力+护理人才+护理人员+助产士+产科人力+产科人才+产科专家+儿科人力+儿科人才+儿科专家+接种人员+疫苗接种者+接生人员+接生人力+接生者+接生婆+医生+护士+大夫)  Translation:  Topic OR Key words= health manpower + health talents + health staff + medical manpower + medical talents + hospital staff+ medical staff+ medical workers+ nursing workforce + nursing talents + nursing staff + pharmaceutical talents + pharmaceutical workforce +[pharmacist](file:///C:\Users\lsh410471\AppData\Local\youdao\dict\Application\7.5.0.0\resultui\dict\?keyword=pharmacist)s + technical talents + technical talents + midwives +[obstetric](file:///C:\Users\lsh410471\AppData\Local\youdao\dict\Application\7.5.0.0\resultui\dict\?keyword=obstetrics) manpower +[obstetric](file:///C:\Users\lsh410471\AppData\Local\youdao\dict\Application\7.5.0.0\resultui\dict\?keyword=obstetrics) talents + obstetric professionals+ paediatric personnel + paediatric talents + paediatric professionals + vaccination personnel + vaccination staff + midwifery personnel + midwifery workforce +midwifery workers + howdie + doctor + nurse + physician | 230185 |
| 2 | MCH services | 题名或关键词:(妇幼保健+妇幼服务+妇幼健康+妇幼卫生+孕期卫生服务+孕产期保健+孕产妇+围产期保健+围生保健+剖腹产+剖宫产+分娩方式+产科护理+住院分娩+孕期保健+孕前保健+产前保健+孕前护理+产前护理+产前检查+儿童健康+儿科护理+儿童保健+婴儿卫生服务+新生儿保健+新生儿护理+新生儿健康+接种+疫苗+计划免疫+免疫规划)  Translation:  Topic OR Key words= maternal and child service + maternal and child healthcare + maternal and child health + maternal and child hygiene + pregnancy health care + [perinatal](file:///C:\Users\lsh410471\AppData\Local\youdao\dict\Application\7.5.0.0\resultui\dict\?keyword=perinatal)health [care](file:///C:\Users\lsh410471\AppData\Local\youdao\dict\Application\7.5.0.0\resultui\dict\?keyword=care) + pregnant women+ perinatal health care+ perinatal care+ caesarean section+ cesarean section+ delivery + obstetric care+ hospital delivery+ pregnancy care+ pre-pregnancy health care+ prenatal health care+ pre-pregnancy care+ prenatal care+ prenatal test+ health care card+ child health+ pediatric care+ child health care+ infant health care+ neonatal care + neonatal health + vaccination+ vaccine+ planned immunization + immunization programme | 160883 |
| 3 |  | 1 AND 2 | 6464 |
