# Supplementary material for: A systematic review of the profile and density of the maternal and child health workforce in China
Source: Hum Resour Health. 2021 Oct 9;19:125. doi: 10.1186/s12960-021-00662-4 (PMC8501553; doi:10.1186/s12960-021-00662-4)
Supplement: Supplementary file 2 — Additional file 2. Quality assessment of included studies. [file 12960_2021_662_MOESM2_ESM.docx]

**Additional file 2**

**A2. Quality assessment of included studies**

**A2.1 Quality assessment for studies reporting on MCH workforce profile**

|  | Definition of health workers | Study design | Completeness of data |
| --- | --- | --- | --- |
| Liao et al, 2017 (E) | Health workers who practiced at a single hospital and held certificates issued by the Ministry of Health | Non-random sampling, data source clearly stated | Not given |
| Risk | Low risk | High risk | Unclear risk |
| Fu, 2012 (C) | Not defined | Health bureau data, data source not clearly stated | Not given |
| Risk | Unclear risk | High risk | Unclear risk |
| Chen, 2016 (C) | Not defined | Non-random sampling, data source not clearly stated | Not given |
| Risk | Unclear risk | High risk | Unclear risk |
| Xiao, 2011 (C Thesis) | Not defined | Census, data source clearly stated | 100% |
| Risk | Unclear risk | Low risk | Low risk |
| Wang et al, 2014 (C) | Not defined | Census, data source clearly stated | 100% |
| Risk | Unclear risk | Low risk | Low risk |
| Huang, 2009 (C) | Health workers who worked in the clinical departments | Census, data source clearly stated | 100% |
| Risk | Low risk | Low risk | Low risk |
| Feng, et al, 2012 (C) | Health workers who worked in the clinical departments | Census, data source clearly stated | 100% |
| Risk | Low risk | Low risk | Low risk |
| Lu et al, 2010 (C) | Health workers who provided outpatient, inpatient, emergent or ambulatory obstetric services | Census, data source clearly stated | 100% |
| Risk | Low risk | Low risk | Low risk |
| Zhu et al, 2008 (C) | Not defined | Census, data source clearly stated | 100% |
| Risk | Unclear risk | Low risk | Low risk |
| Guo et al, 2015 (C) | Not defined | Data from previous survey, but methods not stated | Not given |
| Risk | Unclear risk | High risk | Unclear risk |
| Liu, 2010 (C Thesis) | Not defined | Non-random sampling, data source clearly stated | Not given |
| Risk | Unclear risk | High risk | Unclear risk |
| Shao, 2016 (C) | Health workers who worked in internal wards | Online survey to health workers, but methods not stated | Not given |
| Risk | Low risk | High risk | Unclear risk |
| Yang, 2017 (C Thesis) | Not defined | Cross-sectional survey, but sampling methods not clearly stated | Not given |
| Risk | Unclear risk | High risk | Unclear risk |
| Liu, 2013 (C Thesis) | Health workers who provided clinical services at the frontline | Cross-sectional survey, but sampling methods not clearly stated | 68.75%, no comparison of persons in the study to those not in the study |
| Risk | Low risk | High risk | High risk |
| Sun et al, 2014 (C) | Not defined | Multistage sampling, but methods not clearly stated | Not given |
| Risk | Unclear risk | High risk | Unclear risk |
| Zheng, 2015 (C Thesis) | Health workers who provided child health care | Non-random sampling, data source clearly stated | Not given |
| Risk | Low risk | High risk | Unclear risk |
| Wu et al, 2017 (C) | Paediatric nurse, but no definition | Census, data reported by hospital administrator | 100% |
| Risk | High risk | Low risk | Low risk |
| Li et al, 2014 (C) | Health workers who held midwifery qualifications, either full-time or part-time | Census, data source clearly stated | 100% |
| Risk | Low risk | Low risk | Low risk |
| Ge et al, 2010 (C) | Full-time midwife staff | Non-random sampling, data source clearly stated | Not given |
| Risk | Low risk | High risk | Unclear risk |
| Wang, 2012 (C Thesis) | Health workers who (1) held nursing certificate and qualification for MCH care; (b) working in obstetric department in the past one year; (c) midwifery working experience was more than half a year | Census, data source clearly stated | 88.3% |
| Risk | Low risk | Low risk | Low risk |
| Yu et al, 2015 (C) | Health workers who provided primary health care for children | Census, data source clearly stated | 100% |
| Risk | Low risk | Low risk | Low risk |
| He et al, 1997 (C) | Health workers who provided child healthcare and practiced full-time | Census, data source clearly stated | 80.4% |
| Risk | Low risk | Low risk | Low risk |
| Liao, 2008 (C) | Not defined | Census, data source clearly stated | 100% |
| Risk | Unclear risk | Low risk | Low risk |
| Lu et al, 2013 (C) | Not defined | Census, data source clearly stated | 100% |
| Risk | Unclear risk | Low risk | Low risk |
| Guo et al, 2015 (C) | Not defined | Multi-stage cluster sampling, but methods not clearly stated | Not given |
|  | Unclear risk | Unclear risk | Unclear risk |
| Chen, 1988 (C) | Not defined | Census, data source clearly stated | 100% |
| Risk | Unclear risk | Low risk | Low risk |
| Shen, 1991 (C) | Not defined | Cross-sectional survey, but sampling methods not clearly stated | Not given |
| Risk | Unclear risk | High risk | Unclear risk |
| Zan et al, 2016 (C) | Not defined | Census, data reported by hospital administrator | 100% |
| Risk | Unclear risk | Low risk | Low risk |
| Chen, 2016 (C) | Not defined | Non-random sampling, data reported by hospital administrator | Not given |
| Risk | Unclear risk | High risk | Unclear risk |
| Wang et al, 2014 (C) | Not defined | Census, data source clearly stated | 100% |
| Risk | Unclear risk | Low risk | Low risk |
| Liu et al, 2012 (C) | Health workers who worked in MCH information monitoring and statistics | Census, data source clearly stated | 100% |
| Risk | Low risk | Low risk | Low risk |
| Ye, 1992 (C) | Health workers who provided maternal healthcare in the villages | Cross-sectional survey, but sampling methods not clearly stated | 90% |
| Risk | Low risk | High risk | Low risk |
| Wang, 1975 (E) | Health workers who were selected by the people in the communes and were trained in their locale | Non-random sampling, data source clearly stated | Not given |
| Risk | Low risk | High risk | Unclear risk |
| Cheung et al, 2011 (E) | Health workers who offered midwifery services including all midwives, nurses, doctors, doulas | Non-random sampling, data source clearly stated | 95.3% |
| Risk | Low risk | High risk | Low risk |
| Ren et al, 2015 (E) | Health workers who provided curative and preventive healthcare services and held at least one legal health qualification certificate | Clustered random sampling of health facilities and census of health workers, data source clearly stated | Not given |
| Risk | Low risk | Low risk | Unclear risk |

**A2.2 Quality assessment for studies reporting on MCH workforce density**

|  | Definition of health workers | Study design | Completeness of data |
| --- | --- | --- | --- |
| Ren et al, 2018 (E) | MCH workers who held professional certificates and worked in clinical departments | Nominator from structured questionnaire to health facilities, denominator from local government | Not given |
|  | Low risk | Low risk | Unclear risk |
| Xue et al, 2003 (C) | Not given | Numerator and denominator both from National bureau of statistics | Not given |
|  | Unclear risk | Low risk | Unclear risk |
| Tao et al, 2011 (E) | Midwives with three-year midwifery training | Numerator and denominator both from local health bureau | Not given |
|  | Low risk | Low risk | Unclear risk |
| Hu et al, 2010 (C) | Not given | Numerator from structured questionnaire to health facilities, denominator source not given | Not given |
|  | Unclear risk | Unclear risk | Unclear risk |
| Zhu, 2013 (C) | Not given | Nominator from structured questionnaire to health facilities, denominator from record review of Statistics Yearbook | Not given |
|  | Unclear risk | Low risk | Unclear risk |
| Yang et al, 2016 (C) | Not given | Nominator from structured questionnaire to health facilities, denominator from record review of local health report | Not given |
|  | Unclear risk | Low risk | Unclear risk |
| Wang, 2015 (C Thesis) | Not given | Nominator from structured questionnaire to health facilities, denominator from record review of local health report | Not given |
|  | Unclear risk | Low risk | Unclear risk |
| Song et al, 2016 (E) | Not given | Nominator from structured questionnaire to health facilities, denominator source not given | Not given |
|  | Unclear risk | Unclear risk | Unclear risk |
| Chen et al, 2017 (C) | Not given | Numerator from health bureau data, denominator from census | Not given |
|  | Unclear risk | Low risk | Unclear risk |
| Jin, 2016 (C Thesis) | Not given | Numerator and denominator both from local health bureau | Not given |
|  | Unclear risk | Low risk | Unclear risk |
| Zhang et al, 2019 (E) | Physicians certified by the National Health Commission and licensed as specializing in medical care for children | Nominator from structured questionnaire to health facilities, denominator source not given | The complete rate for the numerator is 91.8%, unclear for denominator |
|  | Low risk | Unclear risk | Unclear risk |
| Ji et al, 2017 (C) | Not given | Numerator from health bureau data, denominator from statistics yearbook | Not given |
|  | Unclear risk | Low risk | Unclear risk |
| Hu et al, 2014 (E) | Full-time vaccination personnel and part-time public health workers who work in childhood immunization | Numerator from health bureau data, denominator source not given | Not given |
|  | Low risk | Unclear risk | Unclear risk |
| Guo et al, 2015 (C) | Public health workers who provided MCH services | Numerator from structured questionnaire to health facilities, denominator from local health bureau | Not given |
|  | Low risk | Low risk | Unclear risk |
| Zou et al, 2016 (C) | Public health workers who provided maternal care or child care | Numerator from record review of local health report, denominator source not given | Not given |
|  | Low risk | Unclear risk | Unclear risk |
| Chen et al, 2010 (C) | Vaccinators who provided outpatient immunization services for children | Numerator from health bureau data, denominator source not given | Not given |
|  | Low risk | Unclear risk | Unclear risk |
| Wang, 1975 (E) | Traditional birth attendants or barefoot doctors who worked on prenatal check-ups, postnatal care or child care in the communes | Numerator from structured interviews of health workers, denominator source not given | Not given |
|  | Low risk | Unclear risk | Unclear risk |
