# Supplementary material for: A systematic review of the profile and density of the maternal and child health workforce in China
Source: Hum Resour Health. 2021 Oct 9;19:125. doi: 10.1186/s12960-021-00662-4 (PMC8501553; doi:10.1186/s12960-021-00662-4)
Supplement: Supplementary file 3 — Additional file 3. Studies reporting on MCH workforce profile: study design and density. [file 12960_2021_662_MOESM3_ESM.docx]

**Additional file 3**

**A3. Studies reporting on MCH workforce profile: study design and density**

Table A3.1. Studies reporting on doctors: study design and profile

| **Study** | **Area (period)** | **Care setting** | | | **Data source** | **Sampling of facilities** | | **Sampling of health workers** | | **Definition of health workers** | | **Sample size of health workers** | | **Educational level** | | **Health-related discipline** |
| --- | --- | --- | --- | --- | --- | --- | --- | --- | --- | --- | --- | --- | --- | --- | --- | --- |
| ***Doctor*** | | |  |  |  |  |  |  |  |  |  |  |  |  |  |  |
| Liao et al, 2017 (E) | 28 provinces, except Shanxi, Hainan, Tibet (2010) | Neonatal units (Undefined) in 61 tertiary hospitals either class A provincial or ministerial affiliated hospitals (20 child hospitals, 29 general hospitals, 12 MCH institutions) | | | Unclear ("reported by hospital administrator") | Unclear (data submitted by facilities as part of "a request for proposals aiming to evaluate national key clinical subspecialty indicators"; sampling method not known) | | Unclear ("not random sampling") | | "Full-time professionals with certificates issued by the Ministry of Health" and "practice at a single hospital (dual practice excluded)". Excluded those in training and excluded "physician assistants, respiratory therapists, physiotherapists, pharmacists, and nutritionists" | | 1369 | | Doctoral (M.D./PhD) degree 20.4% (279); Master degree 50.1% (686); Bachelor degree 29.5% (404) | | _ |
| Fu, 2012 (C) | Yunnan province, autonomous prefecture of Chuxiong (2008) | Units (Undefined) in MCH institutions (excluding prefecture-level institution; number not given) | | | Unclear ("Health bureau data") | Census | | Census | | Undefined | | 229 | | Bachelor degree or above 22.3% (51); Junior college 48.0% (110); Secondary technical school 27.5% (63); High school 1.7% (4); Middle school 0.4% (1) | | _ |
| Chen, 2016 (C) | Fujian province (2015) | Units (Undefined) in 1 MCH institution (provincial level) | | | Unclear ("reported by hospital administrator") | Non-random sampling | | Census | | Undefined | | 408 | | Master degree or above 31.6%; Bachelor degree 64.2%; Junior college or below 4.2% | | _ |
| Xiao, 2011 (C Thesis) | Sichuan province, randomly selected 20 out of 60 counties (2008) | Units (Undefined) in 20 MCH institutions (county level) | | | Structured questionnaire to health workers | Census | | Census | | Undefined | | Undefined | | Master degree or above 0.0%; Bachelor degree 14.3%; Junior college 40.1%; Secondary technical school 45.6% (number not given) | | _ |
| Wang et al, 2014 (C) | Guangxi province, 39 poor counties (2012) | Units (Undefined) in 39 MCH institutions (county level) | | | Structured questionnaire to health workers | Census | | Census | | Undefined | | 1105 | | Master degree or above 0.2% (2); Bachelor degree 22.9% (253); Junior college 58.4% (646); Secondary technical school or below 18.5% (204) | | _ |
| ***Obstetrician or gynaecologist*** | | | |  | | |  | |  | |  | |  | |  |  |
| Huang, 2009 (C) | Guangxi province, Baise city (2008) | Obstetrics or gynaecology department (Undefined) in 150 township hospital (Undefined) | | | Structured questionnaire to health workers | Census | | Census | | "worked in the clinical departments” | | 321 | | Bachelor degree or above 5.9% (19); Junior college 42.4% (136); Secondary technical school 51.7% (166) | | _ |
| Feng, et al, 2012 (C) | Guangxi province, 7 ethnic minority counties (2010) | Units (Undefined) in 112 county hospital and township hospital (Undefined) | | | Structured questionnaire to health workers | Census | | Census | | "worked in the clinical departments” | | 320 | | Bachelor degree 16.3% (52); Junior college 27.5% (88); Secondary technical school 56.3% (180) | | Clinical medicine (Undefined) 80.3% (257); Maternal and child healthcare (Undefined) 18.8% (60); Others (Undefined) 0.9% (3) |
| ***Obstetrician only*** | | | |  | | |  | |  | |  | |  | |  |  |
| Lu et al, 2010 (C) | Jiangxi province, Nanchang city (2008) | Outpatient, inpatient and emergency wards (Undefined) in 98 health facilities “capable of providing obstetric services" | | | Structured questionnaire to health workers | Census | | Census | | "provided outpatient, inpatient, emergent or ambulatory obstetric services” | | 369 | | Bachelor degree or above 39.8%; Junior college 33.9%; Secondary technical school 26.3% (number not given) | | _ |
| Zhu et al, 2008 (C) | Shanghai (2006) | Obstetrics department (including inpatient, outpatient and emergency ward) in 3 MCH institution (tertiary level) | | | Structured questionnaire to health workers | Census | | Census | | Undefined | | 83 | | Postgraduate degree 41.0% (34); Bachelor degree 57.8% (48); Junior college 1.2% (1); Secondary technical school or below 0.0% (0) | | _ |
|  |  | Obstetrics department (including inpatient, outpatient and emergency ward) in 14 general hospitals (tertiary level) | | | *Same as above* | *Same as above* | | *Same as above* | | *Same as above* | | 194 | | Postgraduate degree 40.2% (78); Bachelor degree 58.8% (114); Junior college 0.5% (1); Secondary technical school or below 0.5% (1) | | _ |
|  |  | Obstetrics department (including inpatient, outpatient and emergency ward) in 9 MCH institutions (secondary level) | | | *Same as above* | *Same as above* | | *Same as above* | | *Same as above* | | 164 | | Master degree or above 6.1% (10); Bachelor degree 76.2% (125); Junior college 14.0% (23); Secondary technical school or below 3.7% (6) | | _ |
|  |  | Obstetrics department (including inpatient, outpatient and emergency ward) in 43 general hospitals (secondary level) | | | *Same as above* | *Same as above* | | *Same as above* | | *Same as above* | | 616 | | Master degree or above 6.0% (37); Bachelor degree 62.4% (385); Junior college 27.5% (170); Secondary technical school or below 4.1% (25) | | _ |
|  |  | Obstetrics department (including inpatient, outpatient and emergency ward) in 15 primary healthcare institutions (Undefined) | | | *Same as above* | *Same as above* | | *Same as above* | | *Same as above* | | 99 | | Master degree or above 0.0% (0); Bachelor degree 27.7% (27); Junior college 60.4% (60); Secondary technical school or below 11.9% (12) | | _ |
|  |  | Obstetrics department (including inpatient, outpatient and emergency ward) in 3 private hospitals (Undefined) | | | *Same as above* | *Same as above* | | *Same as above* | | *Same as above* | | 10 | | Master degree or above 20.0% (2); Bachelor degree 80.0% (8) | | _ |
| ***Paediatrician*** | | |  |  | | |  | |  | |  | |  | |  |  |
| Feng, et al, 2012 (C) | Guangxi province, 7 ethnic minority counties (2010) | Units (Undefined) in 112 county hospital and township hospitals (Undefined) | | | Structured questionnaire to health workers | Census | | Census | | Undefined | | 119 | | Bachelor degree 30.2% (36); Junior college 43.7% (52); Secondary technical school 26.1% (31) | | Clinical medicine (Undefined) 99.2% (118); Others (Undefined) 0.8% (1) |
| Guo et al, 2015 (C) | Guangdong province, Guangzhou city (2011) | Paediatric department (Undefined) in “all of the 48 tertiary, secondary and primary health facilities capable of providing paediatric care in central city” | | | Unclear ("data from previous survey") | Undefined | | Undefined | | Undefined | | 1157 | | Doctoral degree 9.8% (114); Master degree 34.6% (400); Bachelor degree 52.7% (610); Junior college or below 2.9% (33) | | _ |
|  |  | Paediatric department (Undefined) in “all of the 31 tertiary, secondary and primary health facilities capable of providing paediatric care in rural-urban continuum” | | | *Same as above* | *Same as above* | | *Same as above* | | *Same as above* | | 205 | | Doctoral degree 4.8% (10); Master degree 18.4% (38); Bachelor degree 72.7% (149); Junior college or below 4.1% (8) | | _ |
|  |  | Paediatric department (Undefined) in “all of the 23 tertiary, secondary and primary health facilities capable of providing paediatric care in outer suburbs” | | | *Same as above* | *Same as above* | | *Same as above* | | *Same as above* | | 187 | | Master degree 7.0% (13); Bachelor degree 83.4% (156); Junior college or below 9.6% (18) | | _ |
| Liu, 2010 (C Thesis) | Guangdong province (2008) | Units (Undefined) in 21 tertiary hospitals (including general hospitals and MCH centres) | | | Structured questionnaire to health workers | Non-random sampling ("Purposive sampling") | | Census | | Undefined | | 539 | | Doctoral degree 1.7% (9); Master degree 15.4% (83); Bachelor degree 76.3% (411); Junior college or below 2.4% (13); No degree 4.3% (23) | | _ |
|  |  | Units (Undefined) in 32 secondary hospitals (including general hospitals and MCH centres) | | | *Same as above* | *Same as above* | | *Same as above* | | *Same as above* | | 575 | | Doctoral degree 0.2% (1); Master degree 5.7% (33);  Bachelor degree 65.6% (377); Junior college or below 25.9% (149); No degree 2.6% (15) | | _ |
|  |  | Units (Undefined) in 6 primary hospitals (including general hospitals and MCH centres) | | | *Same as above* | *Same as above* | | *Same as above* | | *Same as above* | | 80 | | Doctoral degree 0.0% (0); Master degree 0.0% (0); Bachelor degree 51.3% (41); Junior college or below 48.8% (39); No degree 0.0% (0) | | _ |
| Shao, 2016 (C) | Zhejiang province (2015) | Paediatric department (Undefined) in 265 general hospitals, MCH institutions, specialized paediatric hospitals and primary care institutions (Undefined) | | | Unclear ("online survey to health worker") | Undefined | | Undefined | | "work in internal wards" | | 3662 | | Master degree or above 22.6%; Bachelor degree 73.2%; Junior college 4.2% (number not given) | | _ |
| Yang, 2017 (C Thesis) | Guangdong province, Guangzhou city (2014) | Paediatric department (Undefined) in 110 health facilities “capable of providing paediatric care" | | | Structured questionnaire to health workers | Unclear | | Census | | Undefined | | 2247 | | Doctoral degree 4.8%; Master degree 23.2%; Bachelor degree 65.1%; Junior college or below 6.9% (number not given) | | _ |

Table A3.2. Studies reporting on nurses and midwives: study design and profile

| **Study** | **Area (period)** | **Care setting** | **Data source** | **Sampling of facilities** | **Sampling of health workers** | **Definition of health workers** | **Sample size of health workers** | **Educational level** | **Health-related discipline** |
| --- | --- | --- | --- | --- | --- | --- | --- | --- | --- |
| ***Nurse*** |  |  |  |  |  |  |  |  |  |
| Liao et al, 2017 (E) | 28 provinces, except Shanxi, Hainan, Tibet (2010) | Neonatal units (Undefined) in 61 tertiary hospitals either class A provincial or ministerial affiliated hospitals (20 child hospitals, 29 general hospitals, 12 MCH institutions) | Unclear ("reported by hospital administrator") | Unclear (data submitted by facilities as part of "a request for proposals aiming to evaluate national key clinical subspecialty indicators"; sampling method not known) | Unclear ("not random sampling") | "full-time professionals with certificates issued by the Ministry of Health" and "practice at a single hospital (dual practice excluded)". Excluded those "in training such as interns, trainee nurses, fellows, and ward care workers." | 3443 | Doctoral degree 0.1% (2); Master degree 1.2% (40); Bachelor degree 36.9% (1272); Junior college 61.8% (2129) | _ |
| Fu, 2012 (C) | Yunnan province, autonomous prefecture of Chuxiong (2008) | Units (Undefined) in MCH institutions (excluding prefecture-level institution; number not given) | Unclear ("Health bureau data") | Census | Census | Undefined | 119 | Bachelor degree or above 8.4% (10); Junior college 59.7% (71); Secondary technical school 24.4% (29); High school 6.7% (8); Middle school 0.8% (1) | _ |
| Feng, et al, 2012 (C) | Guangxi province, 7 ethnic minority counties (2010) | Units (Undefined) in county hospitals and 112 township hospitals (Undefined) | Structured questionnaire to health workers | Census | Census | "worked in the obstetrics or gynaecology departments” | 337 | Junior college 29.4% (99); Secondary technical school 70.6% (238) | Nursing (Undefined) 99.7% (335); Others (Undefined) 0.3% (1) |
|  |  | *Same as above* | *Same as above* | *Same as above* | *Same as above* | "worked in the paediatric departments” | 135 | Junior college 27.4% (37); Secondary technical school 72.6% (98) | Nursing (Undefined) 100.0% (135) |
| Liu, 2013 (C Thesis) | Hunan province (2012) | Units (Undefined) in 1 MCH institution (Undefined) | Structured questionnaire to health workers | Undefined | Random sampling | "provide clinical services at the frontline" | 275 | Bachelor degree or above 70.9% (195); Junior college 25.1% (69); Secondary technical school or below 4.0% (11) | _ |
| Liu, 2010 (C Thesis) | Guangdong province (2008) | Units (Undefined) in 21 tertiary hospitals (including general hospitals and MCH institutions) | Structured questionnaire to health workers | Non-random sampling ("Purposive sampling") | Census | Unclear (Paediatric nurse) | 726 | Master degree or above 0.1% (1); Bachelor degree 9.4% (68); Junior college 43.7% (317); Secondary technical school 39.8% (289); No degree 7.0% (51) | _ |
|  |  | Units (Undefined) in 32 secondary hospitals (including general hospitals and MCH institutions) | *Same as above* | *Same as above* | *Same as above* | *Same as above* | 840 | Master degree or above 0.0% (0); bachelor degree 4.8% (40); Junior college 40.0% (336); Secondary technical school 53.0% (445); No degree 2.3% (19) | _ |
|  |  | Units (Undefined) in 6 primary hospitals (including general hospitals and MCH institutions) | *Same as above* | *Same as above* | *Same as above* | *Same as above* | 105 | Master degree or above 0.0% (0); Bachelor degree 1.0% (1); Junior college 40.0% (42); Secondary technical school 59.1% (62); No degree 0.0% (0) | _ |
| Sun et al, 2014 (C) | Guangdong, Hainan, Beijing, Jilin, Jiangsu, Shandong and Zhejiang provinces (2012) | Neonatal units (Undefined) in 468 tertiary or secondary hospital (Undefined) | Structured questionnaire to health workers | Unclear ("multistage sampling") | Census | Undefined | 5582 | Master degree or above 1.0% (45); Bachelor degree 39.0% (2190); Junior college 48.0% (2686); Secondary technical schools 12.0% (661) | _ |
| Zheng, 2015 (C Thesis) | Fujian province, Fuzhou city (2014) | Paediatric department (internal wards, surgery wards and intensive care units) in 8 tertiary hospital (capable of providing paediatric service, including 5 general hospitals, 2 specialized children's hospitals and 1 traditional Chinese medicine hospital) | Structured questionnaire to health workers | Unclear ("selected only those with paediatric departments and permanent staff") | Undefined | "provided child health care"; excluding those "have not been formally registered and those who were on leave during the investigation" | 439 | Master degree or above 0.0% (0); Bachelor degree 17.5% (73); Junior college 71.1% (288); Secondary technical school or below 11.4% (44) | _ |
| Yang, 2017 (C Thesis) | Guangdong province, Guangzhou city (2014) | Paediatric department (Undefined) in 110 health facilities “capable of providing paediatric care" | Structured questionnaire to health workers | Unclear | Census | Unclear (Paediatric nurse) | 2820 | Master degree 0.2%; Bachelor degree 29.6%; Junior college 47.6%; Secondary technical school or below 22.6% (number not given) | _ |
| Wu et al, 2017 (C) | Shanghai (2016) | Outpatient, inpatient, emergency and administrative departments, intensive care units, medical laboratory and operating room (Undefined) in 1 tertiary hospital (specialized children's hospital) | Unclear ("reported by hospital administrator") | Census | Census | Unclear (Paediatric nurse) | 665 | Doctoral degree 0.3% (2); Master degree 1.2% (8); Bachelor degree 31.9% (212); Junior college 55.0% (366); Secondary technical school 11.6% (77) | _ |
| Chen, 2016 (C) | Fujian province (2015) | Units (Undefined) in 1 MCH institution (provincial level) | Unclear ("reported by hospital administrator") | Non-random sampling | Census | Undefined | 391 | Master degree or above 0.3%; Bachelor degree 46.3%; Junior college or below 53.5% | _ |
| Xiao, 2011 (C Thesis) | Sichuan province, randomly selected 20 out of 60 counties (2008) | Units (Undefined) in 20 MCH institutions (county level) | Structured questionnaire to health workers | Census | Census | Undefined | Undefined | Master degree or above 0.0%; Bachelor degree 7.8%; Junior college 43.8%; Secondary technical school 48.4% (number not given) | _ |
| Wang et al, 2014 (C) | Guangxi province, 39 poor counties (2012) | Units (Undefined) in 39 MCH institutions (county level) | Structured questionnaire to health workers | Census | Census | Undefined | 1351 | Bachelor degree 3.4% (46); Junior college 49.7% (671); Secondary technical school 46.9% (634) | _ |
| ***Midwives*** | |  |  |  |  |  |  |  |  |
| Li et al, 2014 (C) | Sichuan province, Leshan city (2013) | Units (Undefined) in 75 health facilities “capable of providing obstetric services" (public hospitals at municipal level 3, county level 29, township level 38, private hospital 5) | Structured questionnaire to health workers | Census | Census | "held midwifery qualifications”; included both full-time and part-time staff. | 259 (32 full-time, 227 part-time) | Master degree or above 0.8% (2); Bachelor degree 22.4% (58); Junior college 35.5% (92); Secondary technical school 40.5% (105); High school 0.8% (2) | Midwifery (Undefined) 1.9% (5); Midwifery skill training through continuing training at work 10.5% (27); Others (Undefined) 87.6% (227) |
| Ge et al, 2010 (C) | Shaanxi province (2008) | Units (Undefined) in 68 out of 69 eligible health facilities "capable of providing midwifery services at county or higher level "; excluded MCH institutions at provincial level; excluded health facilities with annual number of natural deliveries less than 100 | Structured questionnaire to health workers | Non-random sampling (Purposive sampling) | Census | Unclear ("Full-time midwife staff") | 367 | Bachelor degree 3.5% (13); Junior college 65.9% (242); Secondary technical school 30.4% (112) | Midwifery (Undefined) 74.9% (275); Nursing (Undefined) 15.0% (55); Clinical medicine (Undefined) 10.1% (37) |
| Wang, 2012 (C Thesis) | Jilin province, all urban districts in Changchun city (2011) | Obstetrics department (Undefined) in 28 health facilities "capable of providing midwifery services and at county level or above" | Structured questionnaire to health workers | Census | Census | “(a) held nursing certificate and qualification for MCH care; (b) working in obstetric department in the past one year; (c) midwifery working experience was more than half an year; (d) not on leave during investigation” | 197 | Master degree or above 11.2% (22); Bachelor degree 52.8% (104); Junior college 26.4% (52); Secondary technical school 9.6% (19) | Midwifery (Undefined) 22.8% (45); Others (Undefined) 77.2% (152) |

Table A3.3. Studies reporting on other cadres: study design and profile

| **Study** | **Area (period)** | | **Care setting** | **Data source** | **Sampling of facilities** | **Sampling of health workers** | **Definition of health workers** | **Sample size of health workers** | **Educational level** | **Health-related discipline** |
| --- | --- | --- | --- | --- | --- | --- | --- | --- | --- | --- |
| ***Specialized public health worker*** | | | | |  |  |  |  |  |  |
| Yu et al, 2015 (C) | | Zhejiang province, Xiaoshan district in Hangzhou city (2009) | Units (Undefined) in 61 township hospital and community health centre (Undefined) | Structured questionnaire to health workers | Census | Census | "provided primary health care for children” | 61 | Junior college or above 75.4% (46); Others (Undefined) | Clinical medicine (Undefined) 100% (61) |
|  | | *Same area as above* (2014) | *Same as above* | *Same as above* | *Same as above* | *Same as above* | *Same as above* | 82 | Junior college or above 90.2% (74); Others (Undefined) 9.8% (8) | Clinical medicine (Undefined) 84.1% (69); Nursing (Undefined) 15.9% (13) |
| He et al, 1997 (C) | | Jiangsu province, Jiang du district (1996) | Units (Undefined) in 41 township hospitals (Undefined) | Structured questionnaire to health workers | Census | Census | "provide child healthcare and practice full-time" | 45 | Secondary technical school 35.6% (16); High school 15.5% (7); Secondary school 48.9% (22) | Public health (Undefined) 35.6% (16); Nursing (Undefined) 22.2% (10); Obstetrics and gynaecology (Undefined) 15.6% (7); Internal medicine (Undefined) 6.7% (3); Paediatrics (Undefined) 2.2% (1); Auxiliary medicine (Undefined) 2.2% (1); Others (Undefined) 15.6% (7) |
| Shao, 2016 (C) | | Henan province (2015) | Paediatric department (Undefined) in 265 general hospitals, maternal and health institutions, specialized paediatric hospitals and primary care institutions (Undefined) | Unclear ("online survey to health workers") | Undefined | Undefined | "provide primary healthcare for children" | 510 | Master degree or above 11.5%; Bachelor degree 69.4%; Junior college or below 19.1% (number not given) | _ |
| Liao, 2008 (C) | | Chongqing municipality, Nanan district (2007) | Units (Undefined) in 1 MCH institution (tertiary level) | Structured questionnaire to health workers | Census | Census | Undefined | 10 | Bachelor degree or above 50.0%; College degree 50.0% (number not given) | _ |
|  | |  | Units (Undefined) in 23 MCH institutions (secondary level) | *Same as above* | *Same as above* | *Same as above* | *Same as above* | 42 | Bachelor degree or above 19.1% (8); Junior college 45.2% (19); Secondary technical school or high school 31.0% (13); Middle school or below 4.7% (2) | _ |
| Lu et al, 2013 (C) | | Zhejiang province, Huzhou (2012) | Units (Undefined) in 67 township hospital and community health centre (Undefined) | Structured questionnaire to health workers | Census | Census | Undefined | 224 | Bachelor degree or above 18.3% (41); Junior college 50.5% (113); Secondary technical school or high school 28.1% (63); Middle school or below 3.1% (7) | _ |
| Guo et al, 2015 (C) | | Zhejiang province, 4 randomly selected counties in Hangzhou (2013) | Units (Undefined) in 62 community health centres (Undefined) | Structured questionnaire to health workers | Unclear ("multi-stage cluster sampling") | Census | Undefined | 205 | Master degree 0.9% (2); Bachelor degree 49.8% (102); Junior college 40.5% (83); Secondary technical school or below 8.8% (18) | Clinical medicine (Undefined) 14.6% (30); Maternal and health care (Undefined) 58.1% (119); Nursing or others (Undefined) 27.3% (56) |
| Chen, 1988 (C) | | Hunan province, all the 25 townships in Hengshan county (1987) | Units (Undefined) in MCH institutions (county level; number not given) | Structured questionnaire to health workers | Census | Census | Undefined | 10 | Junior college 20.0% (2); Secondary technical school 60.0% (6); Middle school 10.0% (1); Primary school 10.0% (1) | _ |
|  | |  | Units (Undefined) in MCH institution (township level; number not given) | *Same as above* | *Same as above* | *Same as above* | *Same as above* | 25 | Junior college 8.7% (2); Secondary technical school 78.3% (20); Middle school 13.0% (3) | _ |
| ***Vaccinator*** | | |  |  |  |  |  |  |  |  |
| Shen, 1991 (C) | Anhui province, rural counties (1990) | | Rural clinics or vaccination posts (Undefined; number not given) | Structured questionnaire to health workers | Unclear | Random sampling | Undefined | 280 | Junior college or secondary technical school 20.4%; Medical training length one year or above without degree 24.3%; Medical training length less than one year 25.4%; Not any training 29.3% (number not given) | Clinical medicine (Undefined) 71.8%; Traditional Chinese medicine (Undefined) 11.1%; Nursing and midwifery (Undefined) 3.2%; other medical profession (Undefined) 13.6%; Nonmedical (Undefined) 0.3% (number not given) |
| Zan et al, 2016 (C) | Zhejiang province, Yinzhou district in Ningbo city (2014) | | Units (Undefined) in 24 child vaccination posts (Undefined) | Unclear ("reported by hospital administrator") | Census | Census | Undefined | 155 | Bachelor degree 70.9% (110); Junior college 15.5% (24); Secondary technical school or below 13.6% (21) | Clinical medicine (Undefined) 10.4% (16); Nursing (Undefined) 27.9% (43); Public health (Undefined) 55.2% (85); Others (Undefined) 6.5% (10) |
| ***Pharmacist*** | | |  |  |  |  |  |  |  |  |
| Chen, 2016 (C) | Fujian province (2015) | | Units (Undefined) in 1 MCH institution (provincial level) | Unclear ("reported by hospital administrator") | Non-random sampling | Census | Undefined | 36 | Master degree or above 19.4%; Bachelor degree 69.4%; Junior college or below 11.1% | _ |
| Xiao, 2011 (C Thesis) | Sichuan province, randomly selected 20 out of 60 counties (2008) | | Units (Undefined) in 20 MCH institutions (county level) | Structured questionnaire to health workers | Census | Census | Undefined | Undefined | Master degree or above 12.5%; Bachelor degree 12.5%; Junior college 12.5%; Secondary technical school 62.5% (number not given) | _ |
| ***Medical technician*** | | |  |  |  |  |  |  |  |  |
| Chen, 2016 (C) | Fujian province (2015) | | Units (Undefined) in 1 MCH institution (provincial level) | Unclear ("reported by hospital administrator") | Non-random sampling | Census | Undefined | 77 | Master degree or above 20.8%; Bachelor degree 54.5%; Junior college or below 24.7% | _ |
| Xiao, 2011 (C Thesis) | Sichuan province, randomly selected 20 out of 60 counties (2008) | | Units (Undefined) in 20 MCH institution (county level) | Structured questionnaire to health workers | Census | Census | Undefined | Undefined | Junior college 42.9%; Secondary technical school or below 57.1% (number not given) | _ |
| Wang et al, 2014 (C) | Guangxi province, 39 poor counties (2012) | | Units (Undefined) in 39 MCH institutions (county level) | Structured questionnaire to health workers | Census | Census | Undefined | 622 | Bachelor degree 9.5% (59); Junior college 45.8% (285); Secondary technical school 44.7% (278) | _ |
| ***Health information worker*** | | | |  |  |  |  |  |  |  |
| Liu et al, 2012 (C) | Jilin province (2009) | | Units (Undefined) in MCH institutions (provincial level; number not given) | Structured questionnaire to health workers | Census | Census | "work in MCH information monitoring and statistics" | 13 | Junior college or above 100% (13) | Clinical medicine (Undefined) 53.8% (7); Health care (Undefined) 0% (0); Nursing (Undefined) 23.1% (3); Computer science (Undefined) 0% (0); Others (Undefined) 23.1% (3) |
|  |  | | Units (Undefined) in MCH institutions (municipal level; number not given) | *Same as above* | *Same as above* | *Same as above* | *Same as above* | 47 | Junior college or above 74.5% (35); Secondary technical school 25.5% (12); High school or below 0% (0) | Clinical medicine (Undefined) 57.5% (27); Health care (Undefined) 23.4% (11); Nursing (Undefined) 10.6% (5); Computer science (Undefined) 0% (0); Others (Undefined) 8.5% (4) |
|  |  | | Units (Undefined) in MCH institutions (county level; number not given) | *Same as above* | *Same as above* | *Same as above* | *Same as above* | 167 | Junior college or above 58.1% (97); Secondary technical school 41.9% (70); High school or below 0% (0) | Clinical medicine (Undefined) 69.5% (116); Health sciences (Undefined) 16.7% (28) Nursing (Undefined) 3.6% (6); Computer science (Undefined) 0.6% (1); Others (Undefined) 9.6% (16) |
|  |  | | Units (Undefined) in 167 township hospitals (Undefined) | *Same as above* | *Same as above* | *Same as above* | *Same as above* | 702 | Junior college or above 25.9% (182); Secondary technical school 70% (491); High school or below 4.1% (29) | Clinical medicine (Undefined) 49.7% (230); Health sciences (Undefined) 20.1% (93); Nursing (Undefined) 17.5% (81); Computer sciences (Undefined) 0.4% (2); Others (Undefined) 12.3% (57) |
| ***Village doctor*** | | | |  |  |  |  |  |  |  |
| Liao, 2008 (C) | Chongqing municipality, Nanan district (2007) | | 67 village clinics (Undefined) | Structured questionnaire to health workers | Census | Census | "practice in the village clinics and provide MCH services” | 67 | Junior college 7.5% (5); High school 58.2% (39); Middle school 29.9% (20); Primary school 4.4% (3) | _ |
| Ye, 1992 (C) | Jiangsu province, Dongtai city (1991) | | 30 village clinics (Undefined) | Structured questionnaire to health workers | Unclear ("random cluster sampling") | Census | “provide maternal health in the villages and are all female” | 588 | Secondary technical school 0.5%; High school 32.3%; Middle school 50.2%; Primary school 17.0% (Number not given) | 3-year medical training 0.5%; 1-year medical training 48.0%; 6-month medical training 43.2%; 3-month medical training 8.3% (Number not given) |
| ***Barefoot doctor*** | | | |  |  |  |  |  |  |  |
| Wang, 1975 (E) | Zhejiang province, one commune named “Four Season Green commune” in the outskirts of Hangzhou (1973) | | 1 commune hospital | Unclear (“interviewed a cross-section of health workers”) | Unclear | Census | Those "selected by the people in the communes and are trained in their locale" and "take on large responsibilities in caring for the health of the mother after birth and the child" | 2 | One-year medical training 50.0%; Four-month medical training 50.0% | - |
| Chen, 1988 (C) | Hunan province, all the 25 townships in Hengshan county (1987) | | NA ("in village") | Structured questionnaire to health workers | Census | Census | "work as part-time birth attendants") | 109 | Junior college 9.2%; Secondary technical school 16.7%; Middle school 44.0%; Primary school 38.5%; No schooling 1.8% | _ |
| ***Traditional birth attendant*** | | | |  |  |  |  |  |  |  |
| Chen, 1988 (C) | Hunan province, all the 25 townships in Hengshan county (1987) | | NA ("in village") | Structured questionnaire to health workers | Census | Census | Undefined | 215 | Secondary technical school 13.0% (28); Middle school 18.5% (40); Primary school 51.8% (111); No schooling 16.7% (36) | _ |
| ***Maternal health worker*** | | | |  |  |  |  |  |  |  |
| Cheung et al, 2011 (E) | Zhejiang Province, 7 cities (2009) | | Maternity units (Undefined) in 9 hospitals (Undefined) | Structured questionnaire to health workers | Unclear ("the units and hospitals were chosen by the researchers because they have personal contacts there") | Unclear ("not random sampling") | “offer midwifery services including all midwives, nurses, doctors, doulas” ) | 241 | _ | Obstetric nursing (Undefined) 75.9% (186); Midwifery (Undefined) 20.8% (51); Maternity/ childcare (Undefined) 1.6% (4); Obstetrics (Undefined) 1.6% (4) |
| Huang, 2009 (C) | Guangxi province, Baise city (2008) | | Obstetrics or gynaecology department (Undefined) in 150 township hospitals (Undefined) | Structured questionnaire to health workers | Census | Census | "work in the preventive medical departments and provide maternal healthcare” | 163 | Bachelor degree or above 3.7% (6); Junior college 27.6% (45); Secondary technical school 68.7% (112) | _ |
| Zhao, 2007 (C) | Xinjiang Uygur Autonomous Region, Xinyuan county (2004) | | Obstetrics or paediatric department (Undefined) in 112 health facilities “capable of providing maternal care at county level or township level” | Structured questionnaire to health workers | Census | Census | "provide frontline maternal health care” | 78 | College degree or above 33.3% (26); Secondary technical school 61.5% (48); Others (Undefined) 5.2% (4) | _ |
| Ren et al, 2015 (E) | 332 randomly selected districts and counties in 27 provinces (2010) | | Units (Undefined) in all general hospitals (Undefined; number not given) | Structured questionnaire to health workers (answered by hospital administrator) | Clustered random sampling ("All the medical and healthcare institutions providing MCH services in randomly selected districts/cities") | Census | "provide curative and preventive healthcare for women and hold at least one legal health qualification certificate." | Undefined | Master degree or above 3.2%; Bachelor degree 32.7%; Junior college 40.9%; High school or below 23.1% (Number not given) | _ |
|  |  | | Units (Undefined) in all MCH institution (Undefined; number not given) | *Same as above* | *Same as above* | *Same as above* | *Same as above* | *Same as above* | Master degree or above 1.4%; Bachelor degree 28.2%; Junior college 38.1%; High school or below 32.4% (Number not given) | _ |
|  |  | | Units (Undefined) in all township health centres/ Community health centres (Undefined; number not given) | *Same as above* | *Same as above* | *Same as above* | *Same as above* | *Same as above* | Master degree or above 0.1%; Bachelor degree 12.3%; Junior college 45.4%; High school or below 42.3% (Number not given) | _ |
|  |  | | Units (Undefined) in all family planning service stations (Undefined; number not given) | *Same as above* | *Same as above* | *Same as above* | *Same as above* | *Same as above* | Master degree or above 0.0%; Bachelor degree 16.2%; Junior college 48.5%; High school or below 35.3% (Number not given) | _ |
| ***Child health worker*** | | | |  |  |  |  |  |  |  |
| Ren et al, 2015 (E) | 332 randomly selected districts and counties in 27 provinces (2010) | | Units (Undefined) in all general hospitals (Undefined; number not given) | Structured questionnaire to health workers (answered by hospital administrator) | Clustered random sampling ("All the medical and healthcare institutions providing MCH services in randomly selected districts/cities") | Census | "provide curative and preventive healthcare for children and hold at least one legal health qualification certificate." | Undefined | Master degree or above 4.6%; Bachelor degree 37.4%; Junior college 39.5%; High school or below 18.4% (Number not given) | _ |
|  |  | | Units (Undefined) in all MCH institutions (Undefined; number not given) | *Same as above* | *Same as above* | *Same as above* | *Same as above* | *Same as above* | Master degree or above 0.9%; Bachelor degree 30.4%; Junior college 41.6%; High school or below 27.1% (Number not given) | _ |
|  |  | | Units (Undefined) in all township health centres/ Community health centres (Undefined; number not given) | *Same as above* | *Same as above* | *Same as above* | *Same as above* | *Same as above* | Master degree or above 0.1%; Bachelor degree 13.6%; Junior college 46.5%; High school or below 39.8% (Number not given) | _ |
|  |  | | Units (Undefined) in all family planning service stations (Undefined; number not given) | *Same as above* | *Same as above* | *Same as above* | *Same as above* | *Same as above* | Master degree or above 0.0%; Bachelor degree 17.6%; Junior college 58.8%; High school or below 23.5% (Number not given) | _ |
