# Supplementary material for: A systematic review of the profile and density of the maternal and child health workforce in China
Source: Hum Resour Health. 2021 Oct 9;19:125. doi: 10.1186/s12960-021-00662-4 (PMC8501553; doi:10.1186/s12960-021-00662-4)
Supplement: Supplementary file 4 — Additional file 4. Meta-analysis for proportions of MCH workers with different education levels. [file 12960_2021_662_MOESM4_ESM.docx]

**Additional file 4**

**A4. Meta-analysis for proportions of MCH workers with different education levels**


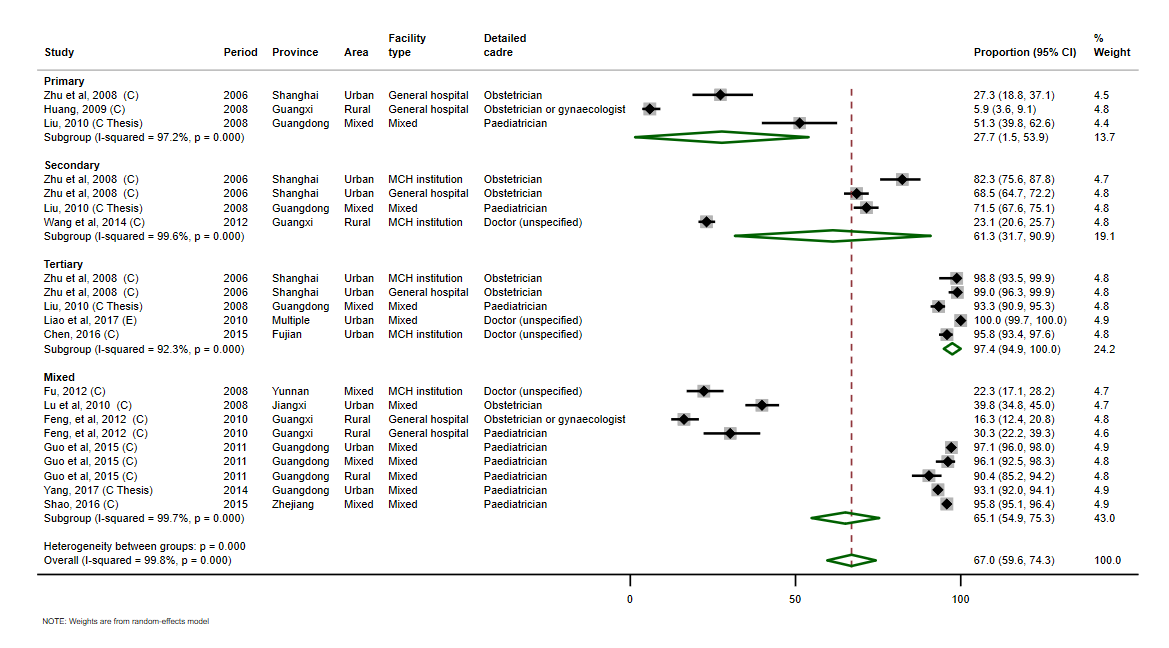


Figure A4.1. Forest plot showing the proportion of doctors holding bachelor or higher-level degrees


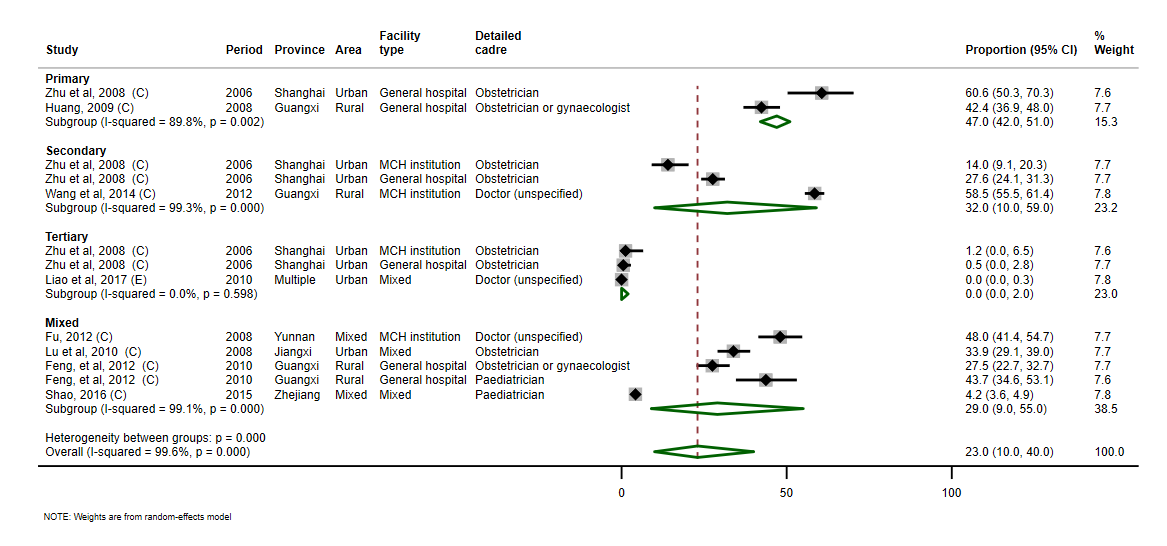


Figure A4.2. Forest plot showing the proportion of doctors holding junior college education


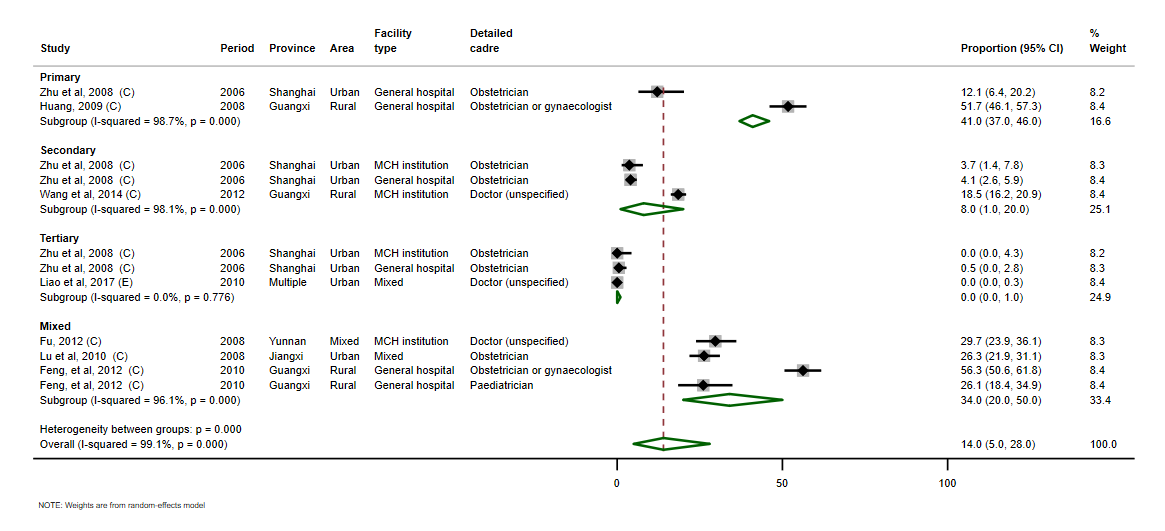


Figure A4.3. Forest plot showing the proportion of doctors with secondary technical school education or below


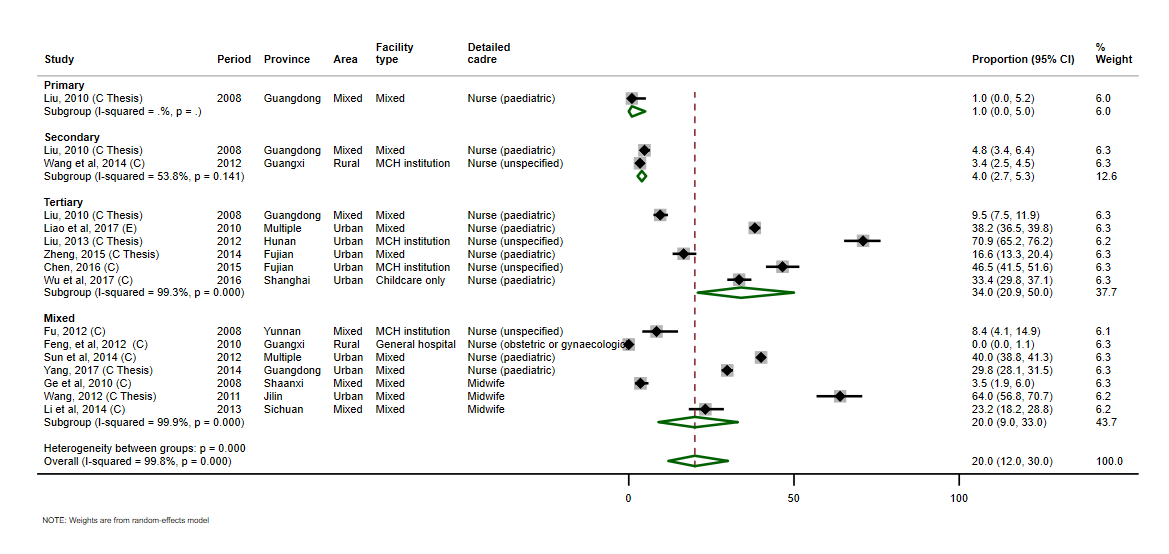


Figure A4.4. Forest plot showing the proportion of nurses (including midwives) holding bachelor or higher-level degrees


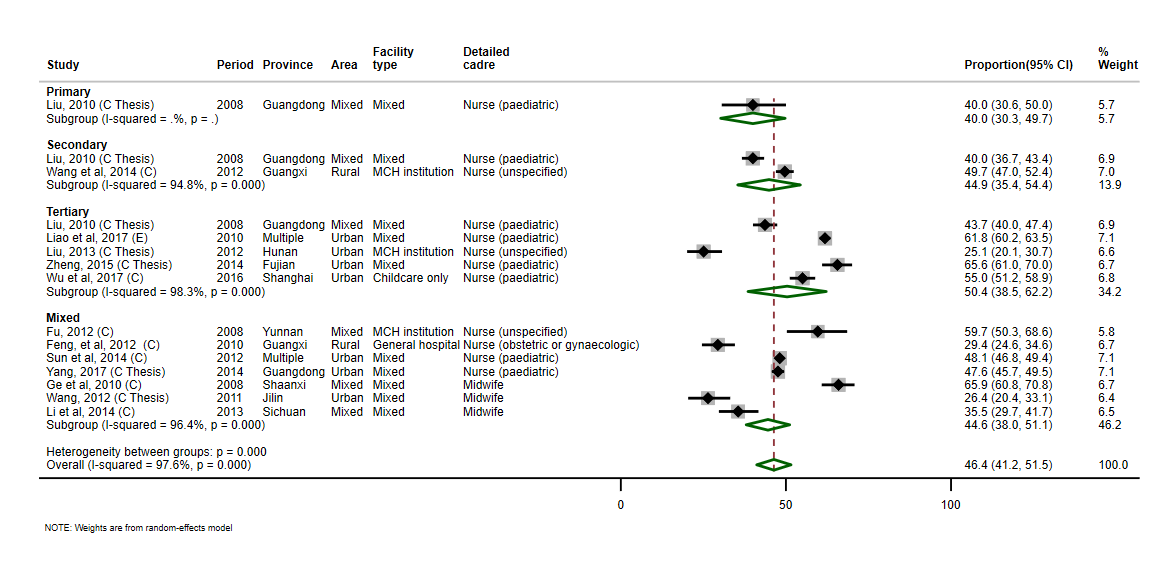


Figure A4.5. Forest plot showing the proportion of nurses (including midwives) holding junior college education


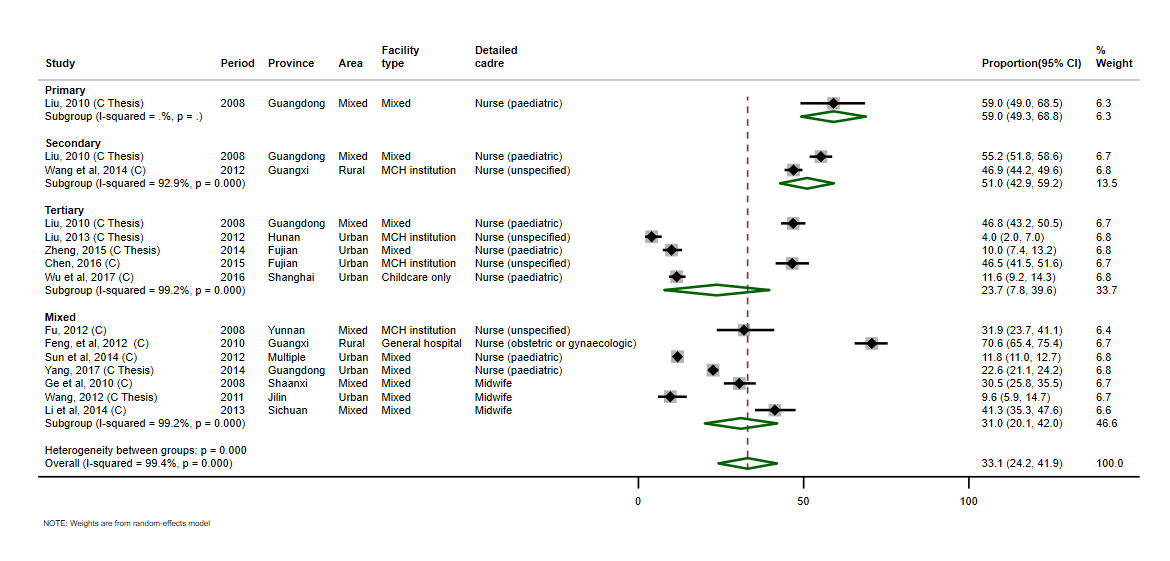


Figure A4.6. Forest plot showing the proportion of nurses (including midwives) with secondary technical school education or below


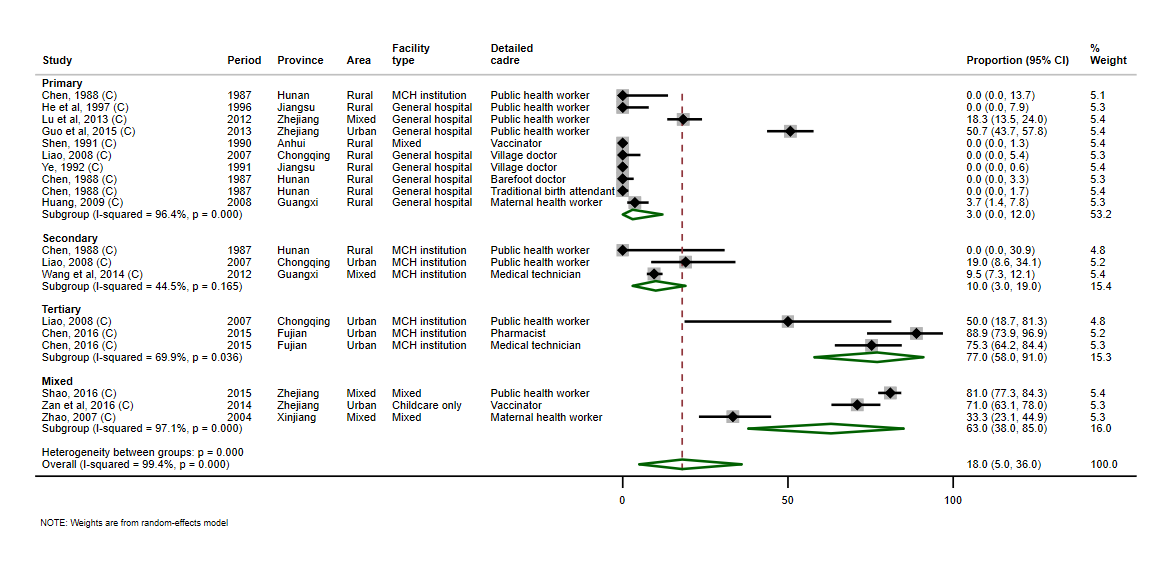


Figure A4.7. Forest plot showing the proportion of other health workers holding bachelor or higher-level degrees


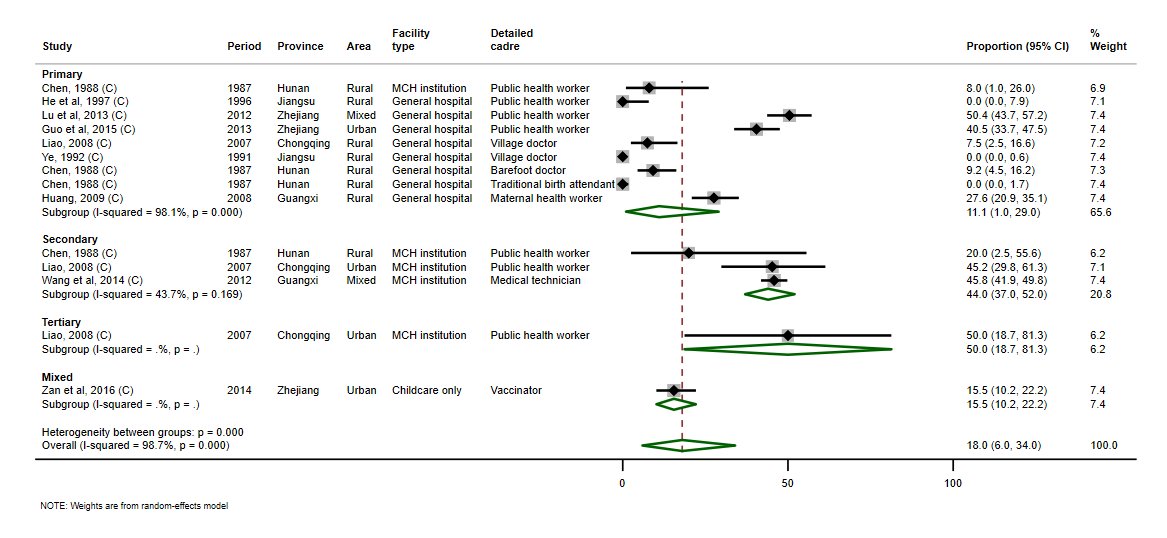


Figure A4.8. Forest plot showing the proportion of other health workers holding junior college education


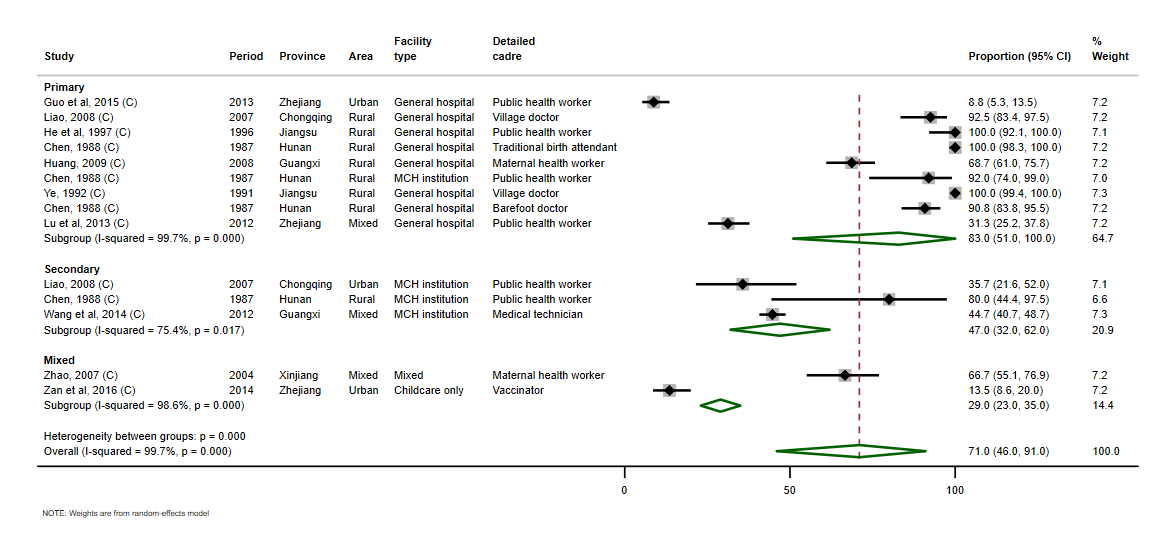


Figure A4.9. Forest plot showing the proportion of other health workers with secondary technical school education or below
