# Supplementary material for: A systematic review of the profile and density of the maternal and child health workforce in China
Source: Hum Resour Health. 2021 Oct 9;19:125. doi: 10.1186/s12960-021-00662-4 (PMC8501553; doi:10.1186/s12960-021-00662-4)
Supplement: Supplementary file 5 — Additional file 5. Studies reporting on MCH workforce density: study design and density. [file 12960_2021_662_MOESM5_ESM.docx]

**Additional file 5**

**A5. Studies reporting on MCH workforce density: study design and density**

Table A5.1. Studies reporting on doctors: study design and density

| **Study** | **Area (period)** | **Care setting** | **Definition of health workers** | **No. of health workers** | **Data source of health worker number** | **Population denominator** | **Size of population** | **Data source of population denominator** | **Density** |
| --- | --- | --- | --- | --- | --- | --- | --- | --- | --- |
| ***Doctor (Obstetrician or gynaecologist)*** | | |  |  |  |  |  |  |  |
| Ren et al, 2018 (E) | 332 randomly selected districts and counties in 27 provinces (2010) | Obstetrics or gynaecology department (Undefined) in all the medical and healthcare institutions providing MCH services, including general hospital, MCH institution, Township health centre, Community health centre, Family planning service station, Other facility (Undefined; number not given) | "work in the obstetric or gynaecology departments” and "hold professional certificates" | 26776 | Structured questionnaire to health facilities (answered by hospital administrator) | Unclear ("total population") | Undefined | Unclear ("from local government") | 2.01 per 10,000 population |
| Xue et al, 2003 (C) | "All the administrative units defined as county representative of the rural areas at national level" (2000) | Undefined | Undefined | Undefined | Unclear (Record review of data collected by Health statistical centre, National bureau of statistics) | Unclear (“total population in rural area”) | Undefined | Unclear (Record review of "National health statistics yearbook") | 0.8 per 10,000 population |
| ***Doctor (Obstetrician)*** | | |  |  |  |  |  |  |  |
| Tao et al, 2011 (E) | Anhui Province, 2 rural counties (2006) | All the 23 facilities in county A and 44 in B including village clinic, township hospital, county hospital (Undefined) | "with three-to-five years of medical training and work in obstetrics” | 82 in county A; 95 in county B | Unclear ("Health bureau data") | Number of women of reproductive age (aged 15-49 years) | 121,483 in county A; 220,187 in county B | Unclear ("data from local health bureau") | 0.67 per 1,000 women of reproductive age in A county;  0.43 per 1,000 women of reproductive age in in B county |
| Chen et al, 2017 (C) | Jiangsu province, Nantong city (2016) | Obstetrics department (Undefined) in all the 97 health facilities "capable of providing obstetrics services" | Undefined | 710 | Unclear ("Health bureau data") | Unclear ("total population") | 7,282,835 | Record review of the “sixth National census” | 0.97 per 10,000 population |
| Hu et al, 2010 (C) | Zhejiang province, Hangzhou city (2007) | Units (Undefined) in all the 70 health facilities "capable of providing obstetrics services" | Undefined | 675 | Structured questionnaire to health facilities | Number of annual live births | 72,098 | Undefined | 9.4 per 1,000 annual live births |
| Ji et al, 2017 (C) | Anhui province (2017) | Units (Undefined) in all the 1358 health facilities "capable of providing obstetrics services" | Undefined | 6798 | Unclear ("Health bureau data") | Number of population actually residing in the area | 61,440,000 | Unclear (Record review of "Anhui statistics yearbook 2015") | 1.11 per 10,000 population |
| Zhu, 2013 (C) | Shanghai (2012) | Units (Undefined) in all health facilities “capable of providing obstetric services" (number not given) | Undefined | 1164 | Structured questionnaire to health facilities | Number of population actually residing in the area | 23,804,300 | Record review of “Shanghai Statistics Yearbook 2012” | 0.49 per 10,000 residential population |
| Yang et al, 2016 (C) | Yunnan province, Kunming city (2014) | Units (Undefined) in all the 125 health facilities “capable of providing obstetric services" | Undefined | 952 | Structured questionnaire to health facilities | Number of annual births | 96,325 | Unclear (Record review of "Annual report of MCH 2014") | 9.9 per 1,000 annual births |
| Wang, 2015 (C Thesis) | Xinjiang province, Yecheng county (2013) | Units (Undefined) in all health facilities “capable of providing obstetric services" (number not given) | Undefined | 62 | Structured questionnaire to health facilities | Number of annual births | Undefined | Unclear (Record review of "Annual report of MCH in Xinjiang 2007-2012") | 6.9 per 1,000 annual births |
|  | Xinjiang province, Shache county (2013) | *Same as above* | *Same as above* | 42 | *Same as above* | *Same as above* | Undefined | *Same as above* | 2.3 per 1,000 annual births |
| ***Doctor (Paediatrician)*** | | |  |  |  |  |  |  |  |
| Song et al, 2016 (E) | 332 randomly selected districts and counties in 27 provinces (2010) | Units (Undefined) in all medical and healthcare institutions providing MCH services, including general hospital, MCH institution, Township health centre, Community health centre, Family planning service station, Other facility (Undefined; number not given) | "provide child healthcare in the frontline” | 16830 | Structured questionnaire to health facilities (answered by hospital administrator) | Unclear ("total population") | Undefined | Undefined | 1.26 per 10,000 population |
| Chen et al, 2017 (C) | Jiangsu province, Nantong city (2016) | Paediatric department (Undefined) in all the 58 health facilities “capable of providing paediatric care” | Undefined | 430 | Unclear ("Health bureau data") | Unclear ("total population") | 7,282,835 | Record review of the “sixth National census” | 0.59 per 10,000 population |
| Jin, 2016 (C Thesis) | Yunnan province, Eshan county (2015) | Paediatric department (Undefined) in all the 2 county hospitals (Undefined) | Undefined | 15 | Unclear ("Health bureau data") | Number of children aged 0 and 14 | 30,200 | Unclear ("data from local health bureau") | 0.49 per 1000 children aged between 0 and 14 |
| Xue et al, 2003 (C) | "All the administrative units defined as county representative of the rural areas at national level" (2000) | Undefined | Undefined | Undefined | Unclear (Record review of data collected by Health statistical centre, National bureau of statistics) | Number of rural population | Undefined | Unclear (Record review of "National health statistics yearbook") | 0.4 per 10,000 population |
| Zhang et al, 2019 (E) | 31 provinces, 2733 counties (2016) | Units (Undefined) in all the 76 children’s hospital, 2184 MCH institutions and 43922 primary hospitals, capable of “providing paediatric care” | “a physician certified by the National Health Commission and licensed as specializing in medical care for children” | 135524 | Structured questionnaire to health facilities (answered by senior hospital manager) | Number of children aged under 14 | Undefined | Undefined | 4 per 10,000 children aged under 14 |

Table A5.2. Studies reporting on nurses and midwives: study design and density

| **Study** | **Area (period)** | **Care setting** | **Definition of health workers** | **No. of health workers** | **Data source of health worker number** | **Population denominator** | **Size of population** | **Data source of population denominator** | **Density** |
| --- | --- | --- | --- | --- | --- | --- | --- | --- | --- |
| ***Nurse*** |  |  |  |  |  |  |  |  |  |
| Song et al, 2016 (E) | 332 randomly selected districts and counties in 27 provinces (2010) | Units (Undefined) in all medical and healthcare institutions providing MCH services, including general hospital, MCH institution, Township health centre, Community health centre, Family planning service station, Other facility (Undefined; number not given) | "provide child healthcare in the paediatric department” | 18134 | Structured questionnaire to health facilities (answered by hospital administrator) | Unclear ("total population") | Undefined | Undefined | 1.36 per 10,000 population |
| Ren et al, 2018 (E) | 332 randomly selected districts and counties in 27 provinces (2010) | Obstetrics or gynaecology department (Undefined) in all medical and healthcare institutions providing MCH services, including general hospital, MCH institution, Township health centre, Community health centre, Family planning service station, Other facility (Undefined; number not given) | "work in the obstetric or gynaecology departments” and "hold professional certificates". | 23465 | Structured questionnaire to health facilities (answered by hospital administrator) | Unclear ("total population") | Undefined | Unclear ("from local government") | 1.76 per 10,000 population |
| Chen et al, 2017 (C) | Jiangsu province, Nantong city (2016) | Obstetrics department (Undefined) in all the 97 health facilities "capable of providing obstetrics services" | Unclear (“Obstetric nurse”) | 968 | Unclear ("Health bureau data") | Unclear ("total population") | 7,282,835 | Record review of the “sixth National census” | 1.33 per 10,000 population |
|  |  | Paediatric department (Undefined) in 58 health facilities “capable of providing paediatric care” | Unclear (“Paediatric nurse”) | 631 | *Same as above* | *Same as above* | 7,282,835 | Record review of the “sixth National census” | 0.86 per 10,000 population |
| Hu et al, 2010 (C) | Zhejiang province, Hangzhou city (2007) | Units (Undefined) in all the 70 health facilities "capable of providing obstetrics services" | Unclear (“Obstetric nurses including midwives") | 1109 | Structured questionnaire to health facilities | Annual live births | 72,098 | Undefined | 15.5 per 1,000 annual births |
| Zhu, 2013 (C) | Shanghai (2012) | Units (Undefined) in all health facilities “capable of providing obstetric services" (number not given) | Unclear (“Obstetric nurse”) | 2032 | Structured questionnaire to health facilities | Number of population actually residing in the area | 23,804,300 | Record review of “Shanghai Statistics Yearbook 2012” | 0.85 per 10,000 residential population |
| Yang et al, 2016 (C) | Yunnan province, Kunming city (2014) | Units (Undefined) in all the 125 health facilities “capable of providing obstetric services" | Unclear (“Obstetric nurse”) | 1597 | Structured questionnaire to health facilities | Number of annual births | 96,325 | Unclear (Record review of "Annual report of MCH 2014") | 16.6 per 1,000 annual births |
| ***Midwives*** |  |  |  |  |  |  |  |  |  |
| Tao et al, 2011 (E) | Anhui Province, 2 rural counties (2006) | All the 23 facilities in A county and 44 facilities in B county including village clinic, township hospital, county hospital (Undefined) | "with three-year midwifery training” | 29 in county A; 56 in county B | Unclear ("Health bureau data") | Number of women of reproductive age | 121,483 in county A; 220,187 in county B | Unclear ("data from local health bureau") | 0.24 per 1,000 women of reproductive age in A county;  0.25 per 1,000 women of reproductive age in in B county; |
| Ren et al, 2018 (E) | 332 randomly selected districts and counties in 27 provinces (2010) | Obstetrics or gynaecology department (Undefined) in all the medical and healthcare institutions providing MCH services, including general hospital, MCH institution, Township health centre, Community health centre, Family planning service station, Other facility (Undefined; number not given) | "work in the obstetric or gynaecology departments” and "hold professional certificates". | 9966 | Structured questionnaire to health facilities (answered by hospital administrator) | Unclear ("total population") | Undefined | Unclear ("from local government") | 0.75 per 10,000 population |
| Ji et al, 2017 (C) | Anhui province (2017) | Units (Undefined) in all the 1358 health facilities "capable of providing obstetrics services" | Undefined | 4674 | Unclear ("Health bureau data") | Number of population actually residing in the area | 61,440,000 | Unclear (Record review of "Anhui statistics yearbook 2015") | 0.77 per 10,000 population |

Table A5.3. Studies reporting on other cadres: study design and density

| **Study** | **Area (period)** | **Care setting** | **Definition of health workers** | **No. of health workers** | **Data source of health worker number** | **Population denominator** | **Size of population** | **Data source of population denominator** | **Density** |
| --- | --- | --- | --- | --- | --- | --- | --- | --- | --- |
| ***Specialized public health worker*** | | |  |  |  |  |  |  |  |
| Hu et al, 2014 (E) | Zhejiang Province, 90 counties (2013) | All the immunization clinic (Undefined; number not given) | "full-time vaccination personnel and part-time public health workers who work in childhood immunization." | Undefined | Unclear ("Health bureau data") | Unclear ("total population") | Undefined | Undefined | 0.13 per 10,000 population (SD 0.28, ranging from 0.03 to 3.74 in different counties) |
| Guo et al, 2015 (C) | Zhejiang province, 4 randomly selected counties in Hangzhou city (2013) | Units (Undefined) in all the 62 community health centres (Undefined) | “provide MCH services” | 205 | Structured questionnaire to health facilities | Population actually residing in the area | 2,570,300 permanent residential population | Unclear ("data provided by surveyed facilities") | 0.8 per 10,000 permanent residential population |
| Zou et al, 2016 (C) | Guangdong province, Guangzhou city (2009) | Units (Undefined) in all community health institutions (Undefined; number not given) | “provide maternal health services” | 297 | Unclear (Record review of "Guangzhou annual report of MCH and healthcare") | Annual number of pregnant women | Undefined | "Annual report of maternal health in Guangzhou 2009" | 3.14 per 1000 pregnant women |
|  | *Same as above* | *Same as above* | “provide child health services” | 321 | *Same as above* | Annual number of children aged between 0 and 6 by actual residence | Undefined | Unclear (Record review of "Annual report of child health (<7 years old) in Guangzhou 2009") | 0.51 per 1000 children aged between 0 and 6 |
|  | Guangdong province, Guangzhou city (2013) | *Same as above* | “provide MCH services” | 404 | *Same as above* | Annual number of pregnant women | Undefined | Unclear (Record review of "Annual report of maternal health in Guangzhou 2013") | 3.38 per 1000 pregnant women |
|  | *Same as above* | *Same as above* | “provide MCH services” | 415 | *Same as above* | Annual number of children aged between 0 and 6 actually residing in the area | Undefined | Unclear (Record review of "Annual report of child health (<7 years old) in Guangzhou 2013") | 0.52 per 1000 children aged between 0 and 6 |
| ***Vaccinator*** |  |  |  |  |  |  |  |  |  |
| Chen et al, 2010 (C) | Guangdong province, Guangzhou city (2003) | All the 174 village clinics (Undefined) | "who provided outpatient immunization services for children” | 939 | Unclear ("Health bureau data") | Unclear ("total population") | 7,272,034 | Undefined | 1.29 per 10,000 population |
|  | *Same area as above* (2009) | All the 210 village clinics (Undefined) | *Same as above* | 1356 | *Same as above* | *Same as above* | 7,989,937 | *Same as above* | 1.70 per 10,000 population |
| ***Barefoot doctor*** | |  |  |  |  |  |  |  |  |
| Wang, 1975 (E) | Liaoning province, Shenyang city (1973) | All the 15 health stations (Undefined) | "selected by the people in the communes and are trained in their locale" and "take on large responsibilities in caring for the health of the mother after birth and the child" | 26 | Unclear ("interviewed the health workers"） | Number of population actually residing in the area | 28,053 | Undefined | 9.3 barefoot doctors per 10,000 population |
| ***Traditional birth attendant*** | | |  |  |  |  |  |  |  |
| Wang, 1975 (E) | Liaoning province, Shenyang city (1973) | All the 15 health stations (Undefined) | "give care and regular check-ups to pregnant women, attend to deliveries at home and give postnatal care to mother and child" | 16 | Unclear ("interviewed the health workers"） | Number of population actually residing in the area | 28,053 | Undefined | 5.7 midwives per 10,000 population |
| ***Maternal and child health worker*** | | |  |  |  |  |  |  |  |
| Ren et al, 2015 (E) | 332 randomly selected districts and counties in 27 provinces (2010) | Units (Undefined) in all the 5168 medical and healthcare institutions providing MCH services, including general hospital, MCH institution, Township health centre, Community health centre, Family planning service station, Other facility (Undefined) | "provide curative and preventive healthcare for women and children and hold at least one legal health qualification certificate." | 77248 | Structured questionnaire to health facilities (answered by hospital administrator) | Unclear ("total population") | Undefined | Undefined | 5.5 per 10,000 population at national level (5.7 for the east, 5.7 for the west regions, and 5.1 for the central region) |
| ***Maternal health worker*** | | |  |  |  |  |  |  |  |
| Ren et al, 2015 (E) | 332 randomly selected districts and counties in 27 provinces (2010) | Units (Undefined) in all the medical and healthcare institutions providing MCH services, including general hospital, MCH institution, Township health centre, Community health centre, Family planning service station, Other facility (Undefined; number not given) | "provide curative and preventive healthcare for women and hold at least one legal health qualification certificate." | 315382 | Structured questionnaire to health facilities (answered by hospital administrator) | Unclear ("total population") | Undefined | Undefined | 2.23 per 10,000 population at national level (2.13 for the east, 2.31 for the west regions, and 2.24 for the central region) |
| ***Child health worker*** | | |  |  |  |  |  |  |  |
| Ren et al, 2015 (E) | 332 randomly selected districts and counties in 27 provinces (2010) | Units (Undefined) in all the medical and healthcare institutions providing MCH services, including general hospital, MCH institution, Township health centre, Community health centre, Family planning service station, Other facility (Undefined; number not given) | "provide curative and preventive healthcare for children and hold at least one legal health qualification certificate." | 19853 | Structured questionnaire to health facilities (answered by hospital administrator) | Unclear ("total population ") | Undefined | Undefined | 1.41 per 10,000 population at national level (1.21 for the east, 1.76 for the west regions, and 1.33 for the central region) |
